# Systematic Review of Safety and Efficacy of Second- and Third-Generation CD20-Targeting Biologics in Treating Immune-Mediated Disorders

**DOI:** 10.3389/fimmu.2021.788830

**Published:** 2022-02-02

**Authors:** Celine Kaegi, Benjamin Wuest, Catherine Crowley, Onur Boyman

**Affiliations:** ^1^ Department of Immunology, University Hospital Zurich, Zurich, Switzerland; ^2^ Faculty of Medicine, University of Zurich, Zurich, Switzerland

**Keywords:** obinutuzumab, ocrelizumab, ofatumumab, ublituximab, veltzumab, immune-mediated diseases, systemic lupus erythematosus, multiple sclerosis

## Abstract

**Background:**

B cells can contribute to immune-mediated disorders. Targeting CD20 has proved to be efficacious in several B cell-mediated immunopathologies, as illustrated by the use of rituximab, the first anti-CD20 monoclonal antibody (mAb). Following rituximab, second- and third-generation anti-CD20 mAbs have been developed and tried in immune-mediated diseases, including obinutuzumab, ocrelizumab, ofatumumab, ublituximab, and veltuzumab. However, their safety and efficacy has not been systematically reviewed.

**Objective:**

To evaluate safety and efficacy of obinutuzumab, ocrelizumab, ofatumumab, ublituximab, and veltuzumab for the treatment of immune-mediated disorders compared to placebo, conventional treatment or other biologics.

**Methods:**

The PRISMA checklist guided the reporting of the data. We searched the PubMed database between 4 October 2016 and 22 July 2021 concentrating on immune-mediated disorders.

**Results:**

The literature search identified 2220 articles. After screening titles and abstracts against the inclusion and exclusion criteria and assessing full texts, 27 articles were finally included in a narrative synthesis.

**Conclusions:**

Obinutuzumab has shown promising results in a case series of patients with phospholipase A_2_ receptor-associated membranous nephropathy and mixed results in systemic lupus erythematosus. Ocrelizumab has been approved for the use in patients with relapsing-remitting multiple sclerosis and primary progressive multiple sclerosis. Ocrelizumab was also tested in patients with rheumatoid arthritis, demonstrating promising results, and in systemic lupus erythematosus, revealing mixed results; however, in these conditions, its use was associated with increased risk of serious infections. Ofatumumab received approval for treating patients with relapsing-remitting multiple sclerosis. Moreover, ofatumumab showed promising results in patients with anti-neutrophil cytoplasmic antibody-associated vasculitis, rheumatoid arthritis, and systemic lupus erythematosus, as well as mixed results in phospholipase A_2_ receptor-associated membranous nephropathy. Ublituximab was assessed in relapsing-remitting multiple sclerosis and neuromyelitis optica spectrum disorder, with promising results, however, the included number of patients was too small to conclude. Veltuzumab was tested in patients with immune thrombocytopenia resulting in improved platelet counts.

**Systematic Review Registration:**

https://www.crd.york.ac.uk/prospero/, identifier CRD4201913421.

## Introduction

Most polygenic immune-mediated disorders, including autoimmune and chronic-inflammatory diseases, result from an imbalance of activating versus regulatory immune effector pathways (1). In certain autoimmune diseases, such as multiple sclerosis (MS), rheumatoid arthritis (RA), and systemic lupus erythematosus (SLE), such immune dysregulation is characterized by activated B cell responses. Dysregulated B cell responses can result in the production of autoantibodies, as typically seen in SLE and RA, or they can contribute to activation of autoreactive T cells without evidence of autoantibody production, as observed in MS (2). Traditional therapies of immune-mediated disorders, including B cell-mediated autoimmune diseases, consisted in the use of corticosteroids (also termed glucocorticoids) and immunosuppressive drugs. However, the long-term application of these treatments is hampered by an increased risk of severe infections and cutaneous malignancies as well as by corticosteroid-mediated side effects (3, 4). Starting in the 1990s, the introduction of biological agents (also called biologics or biologicals) has revolutionized the treatment of allergic, autoimmune and chronic-inflammatory disorders (5, 6). The advantage of biologics stems from their precise targeting of specific molecules, which in turn minimizes unwanted damage to off-target tissues and cells.

Also B cell-mediated immunopathologies have greatly benefitted from the advent of biologics, including monoclonal antibodies (mAbs) targeting different B cell surface molecules or survival factors of B cells (7–9). B cells can contribute to immune-mediated diseases by secreting autoantibodies, acting as antigen-presenting cells, producing cytokines, and forming ectopic lymphoid tissues (2, 10, 11). Targeting the antigen cluster of differentiation 20 (CD20) has proved to be efficacious in several B cell-mediated pathologies, as illustrated by the use of rituximab (RTX), the first anti-CD20 mAb (7, 12). Following RTX, second- and third-generation anti-CD20 mAbs have been developed, including ibritumomab tiuxetan, obinutuzumab (OBI), ocaratuzumab, ocrelizumab (OCR), ofatumumab (OFA), tositumomab, ublituximab (UBL), and veltuzumab (VEL). Notably, most of these anti-CD20 mAbs have initially been generated for the treatment of B cell malignancies (12).

CD20 is a cell surface molecule present as homodimers or homotetramers, which is expressed on B cells starting at the pre-B cell stage, whereas its expression is lost during B cell differentiation into plasmablasts and plasma cells (12–14). CD20 is thought to regulate calcium (Ca^2+^) influx into B cells downstream of the B cell receptor. CD20-targeting mAbs act by depleting all CD20^+^ B cell subsets, while sparing pro-B cells, plasmablasts and plasma cells (14). Thus, administration of RTX rapidly reduces the counts of circulating B cells (15), whereas tissular B cells and antibody-producing B cells are affected to a lesser extent by RTX treatment (16). Repeated use of RTX can result in hypogammaglobulinemia by decreasing serum concentrations of immunoglobulin G (IgG), particularly, when it is used in combination with other immunosuppressive agents, such as high doses of corticosteroids and mycophenolate mofetil (MMF) (17).

B cell depletion by CD20-targeting mAbs is thought to be the result of several mechanisms, such as direct apoptosis of the targeted B cells, complement-dependent cytotoxicity (CDC) of B cells, and fragment crystallizable (Fc) receptor-mediated effector functions, including antibody-dependent cellular cytotoxicity and antibody-dependent cellular phagocytosis of B cells (12). Different anti-CD20 mAbs preferentially employ different mechanisms of B cell depletion and modulation of CD20 molecules, with type I mAbs resulting in the redistribution of CD20 into lipid rafts and internalization, whereas type II mAbs do not appear to cause clustering of CD20 with CD20 remaining on the cell surface. Thus, the type I mAbs RTX, ocaratuzumab, OCR, OFA, UBL and VEL lead to compartmentalization of CD20 into lipid rafts and high CDC activity (12). Conversely, the type II mAbs OBI, ibritumomab tiuxetan, and tositumomab show no or little CD20 clustering and CDC activity, but instead they cause very efficient apoptosis of targeted B cells as well as antibody-dependent cellular cytotoxicity and antibody-dependent cellular phagocytosis (18–20). In addition to its type II modality, OBI was glycoengineered to abrogate a fucose sugar residue in the Fc region, which limits its binding to complement and enhances its affinity for activating Fc γ receptors on natural killer cells and neutrophils, thus causing more efficient antibody-dependent cellular cytotoxicity of both malignant B cells and B cells from RA and SLE patients, compared to RTX (21, 22). Notably, CD20^+^ B cells bind twice as many type I anti-CD20 mAb molecules per cell compared to type II mAbs, which is likely due to different binding modes of these mAbs (13, 23). When bound to CD20, type I mAbs form “seeding” complexes that allow the recruitment of further IgG or CD20 molecules, thus favoring efficient complement activation, whereas type II mAbs interacting with CD20 result in “terminal” complexes that prevent the association of additional type II mAbs and complement components (23).

In a previous publication, we systematically reviewed the safety and efficacy of RTX (7). The anti-CD20 mAbs OBI, OCR, OFA, UBL and VEL have been tried in immune-mediated diseases. Conversely, the murine mAbs ibritumomab tiuxetan and tositumomab, which are conjugated to radioactive yttrium-90 and iodine-131, respectively, have so far only been assessed in patients with B cell malignancies. Similarly, ocaratuzumab has been solely tested in patients with B cell malignancies. In the present article, we provide a systematic review of the current available studies assessing the safety and efficacy of the second- and third-generation anti-CD20 mAbs OBI, OCR, OFA, UBL and VEL in immune-mediated diseases.

## Methods

### Study Design and Protocol Registration

The PRISMA checklist (**Table 1**) guided the reporting of this systematic review (24). We initially registered OCR and VEL on PROSPERO, and subsequently updated our protocol to also include OBI, ocaratuzumab, OFA, and UBL; PROSPERO number CRD42019134321.

**Table 1:** The preferred reporting of systematic reviews and meta-analyses (PRISMA) checklist.

Section/topic	#	Checklist item	Reported on page #
**TITLE**			
Title	1	Identify the report as a systematic review, meta-analysis, or both.	1
**ABSTRACT**			
Structured summary	2	Provide a structured summary including, as applicable: background; objectives; data sources; study eligibility criteria, participants, and interventions; study appraisal and synthesis methods; results; limitations; conclusions and implications of key findings; systematic review registration number.	1
**INTRODUCTION**			
Rationale	3	Describe the rationale for the review in the context of what is already known.	2-3
Objectives	4	Provide an explicit statement of questions being addressed with reference to participants, interventions, comparisons, outcomes, and study design (PICOS).	2-3
**METHODS**			
Protocol and registration	5	Indicate if a review protocol exists, if and where it can be accessed (e.g., Web address), and, if available, provide registration information including registration number.	3
Eligibility criteria	6	Specify study characteristics (e.g., PICOS, length of follow-up) and report characteristics (e.g., years considered, language, publication status) used as criteria for eligibility, giving rationale.	3
Information sources	7	Describe all information sources (e.g., databases with dates of coverage, contact with study authors to identify additional studies) in the search and date last searched.	3
Search	8	Present full electronic search strategy for at least one database, including any limits used, such that it could be repeated.	3
Study selection	9	State the process for selecting studies (i.e., screening, eligibility, included in systematic review, and, if applicable, included in the meta-analysis).	3
Data collection process	10	Describe method of data extraction from reports (e.g., piloted forms, independently, in duplicate) and any processes for obtaining and confirming data from investigators.	3
Data items	11	List and define all variables for which data were sought (e.g., PICOS, funding sources) and any assumptions and simplifications made.	3
Risk of bias in individual studies	12	Describe methods used for assessing risk of bias of individual studies (including specification of whether this was done at the study or outcome level), and how this information is to be used in any data synthesis.	3
Summary measures	13	State the principal summary measures (e.g., risk ratio, difference in means).	3
Synthesis of results	14	Describe the methods of handling data and combining results of studies, if done, including measures of consistency (e.g., I^2^) for each meta-analysis.	3
Risk of bias across studies	15	Specify any assessment of risk of bias that may affect the cumulative evidence (e.g., publication bias, selective reporting within studies).	3
Additional analyses	16	Describe methods of additional analyses (e.g., sensitivity or subgroup analyses, meta-regression), if done, indicating which were pre-specified.	NA
**RESULTS**			
Study selection	17	Give numbers of studies screened, assessed for eligibility, and included in the review, with reasons for exclusions at each stage, ideally with a flow diagram.	4
Study characteristics	18	For each study, present characteristics for which data were extracted (e.g., study size, PICOS, follow-up period) and provide the citations.	4-11
Risk of bias within studies	19	Present data on risk of bias of each study and, if available, any outcome level assessment (see Item 12).	11
Results of individual studies	20	For all outcomes considered (benefits or harms), present, for each study: (a) simple summary data for each intervention group (b) effect estimates and confidence intervals, ideally with a forest plot.	4-11
Synthesis of results	21	Present results of each meta-analysis done, including confidence intervals and measures of consistency.	NA
Risk of bias across studies	22	Present results of any assessment of risk of bias across studies (see Item 15).	NA
Additional analysis	23	Give results of additional analyses, if done (e.g., sensitivity or subgroup analyses, meta-regression [see Item 16]).	NA
**DISCUSSION**			
Summary of evidence	24	Summarize the main findings including the strength of evidence for each main outcome; consider their relevance to key groups (e.g., healthcare providers, users, and policy makers).	11-12
Limitations	25	Discuss limitations at study and outcome level (e.g., risk of bias), and at review-level (e.g., incomplete retrieval of identified research, reporting bias).	12
Conclusions	26	Provide a general interpretation of the results in the context of other evidence, and implications for future research.	12-14
**FUNDING**			
Funding	27	Describe sources of funding for the systematic review and other support (e.g., supply of data); role of funders for the systematic review.	NA

### Search Strategy

We searched the PubMed database and reference lists of included studies for suitable clinical trials. The search was conducted between 4 October 2016 and 22 July 2021 for OCR, VEL, OBI, and OFA. Ocaratuzumab and UBL were added during the revision process of this paper and the search was carried out on the 28^th^ of November. Our full search strategy and research terms were defined in advance (**Table 2**). We also used filters for randomized controlled trials (RCTs). If publications were not available via institutional access or open access, study authors were contacted to receive the article or missing trial information.

**Table 2:** Search terms.

01. obinutuzumab 02. obinutuzumab AND ITP 03. obinutuzumab AND immune 04. obinutuzumab AND thrombocytopenia 05. obinutzumab AND vasculitis	633 1 82 26 3
06. ocrelizumab 07. ocrelizumab AND ITP 08. ocrelizumab AND immune 09. ocrelizumab AND thrombocytopenia	531 0 118 1
10. ocrelizumab AND rheumatoid arthritis 11. ocrelizumab AND rheumatoid arthritis; Filters: Randomized Controlled Trial	33 5
12. ocrelizumab AND multiple sclerosis 13. ocrelizumab AND multiple sclerosis; Filters: Randomized Controlled Trial	435 14
14. ocrelizumab AND lupus 15. ocrelizumab AND lupus; Filters: Randomized Controlled Trial	24 2
16. ofatumumab 17. ofatumumab AND lupus 18. ofatumumab AND nephritis 19. ofatumumab AND SLE	627 20 16 8
20. ofatumumab AND multiple sclerosis 21. ofatumumab AND multiple sclerosis; Filters: Randomized Controlled Trial	78 3
22. ofatumumab AND rheumatoid arthritis 23. ofatumumab AND rheumatoid arthritis; Filters: Randomized Controlled Trial	27 3
24. ofatumumab AND vasculitis 25. ofatumumab AND ANCA	7 4
26. tositumomab	348
27. veltuzumab 28. veltuzumab AND pemphigus vulgaris 29. veltuzumab AND pemphigus	51 5 5
30. veltuzumab AND immune thrombocytopenia 31. veltuzumab AND thrombocytopenia	5 5
32. veltuzumab AND multiple sclerosis	1
33. veltuzumab AND rheumatoid arthritis 34. veltuzumab AND arthritis	3 3
35. veltuzumab AND SLE 36. veltuzumab AND lupus	1 4
37. ublituximab	33
38. ocaratuzumab	11

### Eligibility Criteria

We included RCTs, their extension trials and their substudies with predefined endpoints investigating the use of OBI, ocaratuzumab, OCR, OFA, tositumomab, UBL, and VEL in immune-mediated diseases. If RCTs were not available, we included non-randomized clinical studies with at least five patients per intervention group and case series including at least three patients, with the exception of case series stating to be retrospective. We excluded retrospective trials, posthoc-analyses, substudies without predefined endpoints, meta-analyses, reviews, and studies from registries as well as studies carried out on animal models or where the primary endpoint was non-clinical. Trials had to be available in either English or German.

We included primary immune-mediated conditions, including rare diseases. We excluded studies in hematological malignancies and allergic disorders, as they were not within the scope of this article.

### Study Selection, Data Collection Process and Analysis

Three authors (CK, BW, and OB) developed and tested a data extraction sheet, whereupon two authors independently (CK and BW) searched PubMed according to the predefined search terms, checked titles and abstracts, carried out a full-text review of the selected studies, and extracted the relevant data. Any disagreements about study inclusion were resolved by consensus.

### Risk of Bias Assessment

CK used a modified version of the Downs and Black tool (see **Table S1**) to assess the retrieved studies for bias (25). The studies were scored out of a maximum of 28 points for the following categories: (i) reporting, (ii) external validity, (iii) internal validity, and (iv) power, and the scores were summed and ranked high (23-28 points), medium (15-22 points) and low (0-14) quality. Any discrepancies were resolved by consensus.

As we limited our research strategy to the PubMed database, the reference list of these studies, and the expertise of the authors involved, we did not conduct a risk of bias assessment across the studies, as we believed the risk of publication bias was high.

### Principal Summary Measures and Synthesis of Results

The aim of this systematic review was to provide a structured and complete overview of the current available studies assessing the safety and efficacy of OBI, OCR, OFA, tositumomab, UBL, and VEL as well as their influence on quality of life (QoL) when used in immune-mediated diseases. Since we wanted to give an overview we did not specify in more detail these endpoints in order not to exclude potentially important studies.

## Results

### Study Selection and Characteristics

**Figure 1:**
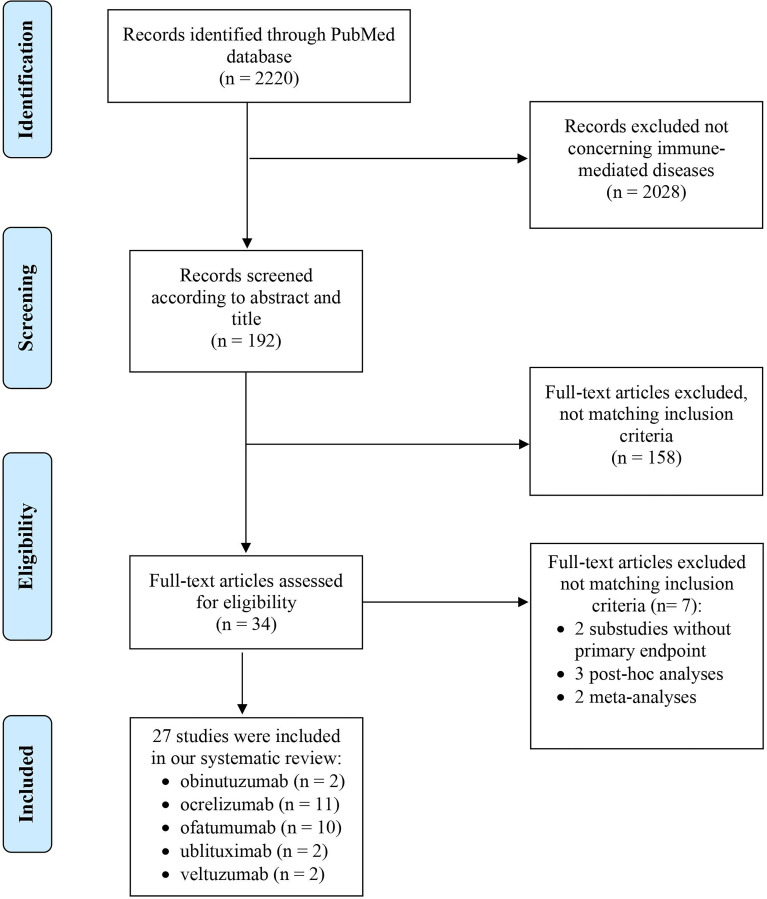
PRISMA diagram of the literature search.

The PubMed search resulted in 2220 articles. We screened 192 of them for title and abstract and, finally, 27 publications were included in the systematic review (**Figure 1**). The main characteristics are available in **Table S2**.

### Synthesized Findings

#### Obinutuzumab

##### Membranous Nephropathy

Our systematic literature search revealed one prospective case series using OBI in three patients with phospholipase A_2_ receptor (PLA_2_R)-mediated membranous nephropathy who had previously been refractory to treatment with RTX (26). The duration of the study was 27 months for the first two cases and 30 months for the third case. The article does not mention the source of funding of the study.

The first patient (case 1, 54-year old white woman) presented with nephrotic syndrome, diagnosed as PLA_2_R-associated membranous nephropathy based on a kidney biopsy. She showed persistently elevated anti-PLA_2_R antibody titers and severe proteinuria despite a treatment with two courses (six months apart) of twice 1 g RTX. Thus, the patient was premedicated with 40 mg intravenous (IV) methylprednisone plus 25 mg oral diphenhydramine and 650 mg oral acetaminophen, followed by treatment with 1 g OBI, given 100 mg IV the first day and 900 mg IV the second day, to reduce possible infusion reactions. 12 and 18 months after treatment with OBI, the patient’s anti-PLA_2_R antibody titers and proteinuria became low and kept on decreasing, respectively, along with an improvement of serum albumin and serum creatinine concentrations.

The second patient (case 2, 61-year old white man) had also nephrotic syndrome, diagnosed as PLA_2_R-associated membranous nephropathy based on a kidney biopsy. He was treated with cyclosporine and prednisone, which resulted in a transient improvement of anti-PLA_2_R antibody titers and proteinuria. Because proteinuria continued to be severe, he was given prednisone and cyclophosphamide, which did not improve the patient’s situation, followed by discontinuation of cyclophosphamide after nine months of treatment and administration of RTX. Despite these treatments, the patient showed an increase in anti-PLA_2_R antibody titers and very severe proteinuria, which motivated a treatment with OBI, given 100 mg IV on day 1, 900 mg IV on day 2, and 1 g IV on day 8, along with a premedication similar to case 1. Seven and nine months after treatment with OBI, the patient’s anti-PLA_2_R antibody titers and proteinuria became low and kept on decreasing, respectively, along with an improvement of serum albumin and serum creatinine concentrations.

The third patient (case 3, 54-year old white man) also presented with nephrotic syndrome, diagnosed as PLA_2_R-associated membranous nephropathy based on a kidney biopsy. He received a treatment with two courses (three months apart) of twice 1 g RTX, following which his anti-PLA_2_R antibody titers decreased, however, his severe proteinuria remained unchanged. Thus, a treatment with OBI was initiated, given 100 mg IV on day 1, 900 mg IV on day 2, and 1 g IV on day 15. Six, 18 and 24 months after receiving OBI, the patient’s anti-PLA_2_R antibody titers remained undetectable and his proteinuria decreased and kept on decreasing, along with an improvement of serum albumin concentrations.

Only one adverse event was noted during treatment with OBI. Patient 3 experienced localized herpes zoster reactivation, which was managed conservatively. There were no other adverse events (AEs) or serious adverse event (SAEs).

The health-related QoL was not assessed.

*Synopsis:* Based on a case series of three patients with PLA_2_R-associated membranous nephropathy whose disease was refractory to treatment with RTX, OBI was more efficacious than RTX in reducing proteinuria and improving serum albumine concentrations. RCTs are needed to confirm these promising results.

##### Systemic Lupus Erythematosus

We found one multicenter double-blind RCT comparing OBI to placebo treatment in 125 patients with SLE and proliferative lupus nephritis (27). All patients received maintenance treatment with MMF and corticosteroids. Furthermore, concomitant treatment with an antimalarial drug, angiotension-converting enzyme inhibitor, angiotensin II receptor blocker, calcium and vitamin D was allowed.

62 patients received placebo and 63 patients OBI. OBI was administered at a dose of 1000 mg on day 1, week 2, week 24, and week 26. The primary endpoint, proportion of patients with complete renal response – measured by urine protein-to-creatinine ratio of less than 0.5, normal serum creatinine and inactive urinary sediment – at 52 weeks was met more often in patients treated with OBI, but the difference was not statistically significant between OBI and placebo (p = 0.115). However, significantly more patients in the OBI group reached an overall renal response (p = 0.025). Although clinical endpoints did not differ markedly between OBI and placebo, patients receiving OBI significantly increased complement factors C3 and C4 and significantly decreased titers of anti-double stranded DNA antibodies.

91% of patients receiving OBI experienced at least one AE and 25% had a SAE. There was one death in the OBI group caused by a gastrointestinal perforation. Urinary tract infection and bronchitis were the most common AEs.

*Synopsis*: This RCT in SLE with active lupus nephritis showed little efficacy of OBI on disease progression when compared to placebo. Further studies with different dosing regimens of OBI are needed to draw a conclusion.

#### Ocrelizumab

##### Multiple Sclerosis

We identified four double-blind placebo-controlled RCTs, one open-label extension study, and one substudy with predefined endpoints using OCR in patients suffering from MS (28–32). Study duration varied from 24 to 192 weeks. All studies were funded by the industry.

In total 2621 patients with relapsing-remitting multiple sclerosis (RRMS) (28, 29) and 732 patients with primary progressive multiple sclerosis (PPMS) (29) were treated with either OCR or a control medication. In three of the studies diagnosis was made based on the McDonald criteria (29–31). Predefined expanded disability status scale (EDSS) had to be between 0 and 6.5 and all patients had to be at least 18 years of age.

In the first study, published in 2011, patients in the active treatment arms received IV OCR (300 mg or 1000 mg) on days 1 and 15 and again on day 1 (600 mg or 1000 mg, respectively) of the second, third, and fourth cycle (weeks 24, 48 and 72) (28). The control group was treated with matching placebo. A fourth treatment arm received open-label interferon-β-1a weekly until week 24. The placebo and interferon-β-1a arms were both offered two doses of OCR (300 mg) on days 1 and 15 of the second, third, and fourth cycle. The OPERA I and II trials used a similar treatment regimen administering 600 mg of IV OCR every 24 weeks compared to interferon β-1a (29). The only study available in patients with PPMS used the same dosing regimen for OCR compared to placebo (29). 100 mg IV methylprednisolone was given as premedication in all four studies. The primary endpoint was either the total number of gadolinium-enhancing (GdE) T1 lesions at weeks 12, 16, 20, and 24, the annualized relapse rate at week 96, the percentage of patients with a disability progression at week 12, or the proportion of infusion-related reactions (IRR). Secondary endpoints comprised the relapse rate, disability progression, proportion of relapse-free patients, safety, as well as various assessments concerning MRI lesions.

The mean number of GdE T1 lesions, the primary endpoint of the study by Kappos et al., decreased significantly as compared to placebo (28). There was an 89% reduction in the 300 mg group (p<0.0001) and a 96% reduction in the 1000 mg group (p<0.0001). Furthermore, the annualized relapse rate was significantly reduced and the total number of new and persisting GdE lesions was significantly lower in both OCR groups.

In the OPERA I and II trials, also conducted in patients with RRMS, there was also a significant reduction (46% and 47%, respectively) in the annualized relapse rate as compared to interferon-β-1a (29). Thus, the primary endpoint was achieved. Furthermore, OCR led to a significant decrease in GdE lesion on T1 MRI and a reduced number of new or newly enlarged T2 lesions. After completion of the double-blind phase, patients could enter an open-label extension trial, where OCR was administered at a dose of 600 mg every 24 weeks (32). The trial was planned for a duration of eight years, with results of the three-year follow-up available currently. Annualized relapse rates remained low in the group previously receiving OCR and continuing to receive OCR during the open-label extension phase. Moreover, there was a significant reduction in annualized relapse rates in the group receiving interferon-β-1a during the double-blind period, followed by OCR during the open-label extension phase. A significant difference between these two groups in terms of mean change in EDSS, brain atrophy, and clinical disease progression remained in the open-label extension phase. No significant differences were noted concerning the number of MRI lesions. Safety data during the extension phase were consistent with the double-blind phase.

Remarkably, results in patients with PPMS were similar to those seen in patients with RRMS. There was a significant reduction in disease progression as early as week 12 (29). The results remained significant until at least week 24. Furthermore, patients in the OCR group had a significantly smaller volume of hyperintense T2 lesions and a significantly smaller change in brain volume.

The ENSEMBLE PLUS substudy in patients with RRMS investigated the occurrence of IRRs in patients receiving OCR at a conventional infusion rate amounting to an infusion time of 3.5 hours versus a shorter infusion time of 2 hours (31). Primary endpoint was the proportion of patients with IRRs following the first dose of OCR. Although there was a slight increase in IRRs in patients receiving the shorter infusion rate, this difference was not significant and there were no serious IRRs in either group. Thus, a shorter infusion rate was considered safe.

AEs and SAEs occurred at a similar frequency in patients treated with OCR, interferon-β-1a, and placebo. Nine patients died during the studies, including two cases of suicide (29), one road-traffic accident (30), one mechanical ileus (29), one pulmonary embolism (30), one pancreatic carcinoma (30), one systemic inflammatory response syndrome of unknown cause (28), one case of pneumonia (30), and one case of aspiration (30). The number of deaths during the open-label extension study was unavailable.

The health-related QoL was assessed in neither of the studies.

*Synopsis:* Above-mentioned trials demonstrated a superiority of OCR above placebo and interferon-β-1a leading to the approval of the drug by the European Medicines Agency (EMA) and the U.S. Food and Drug Administration (FDA) for the use in patients with RRMS and PPMS.

##### Rheumatoid Arthritis

Five placebo-controlled, double-blind RCTs using OCR in patients with RA met our inclusion criteria (33–37). The study durations ranged from 48 to 104 weeks, including two trials, which were terminated early (34, 36).

2835 patients participated in either of the trials. Main inclusion criteria were diagnosis of RA according to the 1987 revised American College of Rheumatology (ACR) criteria and active disease. In most studies a minimum disease duration of three months was required for inclusion. Inflammatory joint disease other than RA and systemic involvement secondary to RA were the most common exclusion criteria (35–37).

OCR was usually given two weeks apart at doses ranging from 10 mg to 1000 mg with either concomitant methotrexate or leflunomide. In all but one trial (37), other disease-modifying antirheumatic drugs (DMARDs) had to be discontinued four weeks prior to enrollment (33–36). Premedication consisted of 100 mg IV methylprednisolone with the exception of the ACTION trial (33). All patients were allowed to use acetaminophen and an antihistamine as premedication (34–37).

Three studies assessed the ACR20 response rate as primary endpoint (34, 35, 37). In contrast, the FILM trial was planned to investigate the change in the van der Heijde-modified total Sharp score at week 104, but due to early termination this endpoint was analyzed earlier at week 52. The ACTION trial (33) analyzed safety events as primary outcome measure. Secondary endpoints comprised ACR50/70 response rates, change in the health assessment questionnaire–disability index, remission rate according to the 28-joint disease activity score (DAS28), and European League Against Rheumatism (EULAR) responses.

Rigby et al. (35), Stohl et al. (36), and Tak et al. (37), reported significant results concerning ACR20, ACR50, and ACR70 response rates as well as DAS28-erythrocyte sedimentation rate (ESR) remission rates. One of the two remaining trials reported significant ACR20 response rates in all OCR-treated patients while ACR50 response rates were only significant in two OCR arms (50 mg and 200 mg) (34). The last trial did not report any *p*-values concerning those endpoints (33).

There was no statistically significant difference in the occurrence of AEs between patients treated with OCR and patients receiving placebo. Although the STAGE and the SCRIPT studies reported comparable frequencies of SAEs and infections, the number of serious infections was elevated in patients receiving OCR leading to the early termination of two other trials (34, 36). In total 8/1951 patients receiving OCR and 5/1007 placebo-treated patients died.

Three studies assessed change in health assessment questionnaire–disability index as a marker for QoL (35–37). All three studies showed a significant improvement.

*Synopsis:* Although OCR led to significantly better results when assessing the ACR response rates as well as the DAS28-ESR remission rates, two studies reported increased rates in serious infections.

##### Systemic Lupus Erythematosus

Only one double-blind, placebo-controlled RCT assessed the efficacy of OCR in patients with SLE (38). The study lasted 96 weeks. Patients with an adequate response at week 48 continued blinded treatment, whereas patients with an inadequate response had the option of open-label treatment.

378 patients were initially enrolled. Diagnosis of SLE according to the ACR criteria with active lupus nephritis class III/IV were the main inclusion criteria. Patients with an eGFR <25ml/min were excluded. Minimum age for inclusion was 16 years.

OCR (400 mg or 1000 mg) was given on days 1 and 15 followed by a single infusion at week 16 and every 16 weeks thereafter. The control group received matching placebo. A premedication consisting of methylprednisolone, acetaminophen, and an antihistamine was given. Furthermore, all patients received concomitant treatment with MMF (3 mg/d) or cyclophosphamide (500 mg IV every 2 weeks for 6 times) followed by a maintenance therapy with azathioprine.

The proportion of patients with a renal response at week 48 was the primary endpoint and was higher in patients receiving OCR. However, the difference was not statistically significant.

83.4% of the patients receiving OCR had at least one AE, which was comparable with the 88% in the placebo group. The percentage of patients with at least one SAE was also comparable, amounting to 28.85% vs 27.2% in the OCR and placebo group, respectively. Remarkably as in patients with RA, the rate of serious infections was increased at 18.2% in patients receiving OCR vs 14.4% in placebo-treated patients, leading to early termination of the study.

Influence on QoL was not assessed.

*Synopsis:* OCR improved the renal response rate, however, this change was not significant when compared to placebo. OCR led to an increased rate of serious infections.

#### Ofatumumab

##### ANCA-Associated Vasculitis

There was only one case series eligible for our review. It tested the efficacy and safety of OFA in patients with ANCA-associated vasculitis (AAV) over a period of 2 years (39). Eight patients with a mean age of 52 years matched the only reported inclusion criteria being a diagnosis of AAV.

IV OFA was given at a dose of 700 mg on days 0 and 14. Concomitant treatment comprised 1 mg/kg oral prednisolone and 10 mg/kg cyclophosphamide, the latter given IV on days 0 and 14 and every 14 days thereafter. After three months, maintenance therapy with azathioprine or MMF was introduced. All patients received prophylactic co-trimoxazole for 3 months, a proton pump inhibitor, and calcium and vitamin D3 supplementation.

There were no predefined endpoints set. All patients achieved clinical remission by month 3. This was accompanied by the ability to taper corticosteroids and by a reduction in acute phase reactants. No relapse occurred during the first year of the study.

Five patients experienced an AE. None of them were considered severe AEs or SAEs.

QoL was not analyzed.

*Synopsis:* Currently available results seem promising, although OFA was only used in eight patients suffering from AAV. Further trials with a randomized-controlled design involving more patients are needed to confirm theses findings.

##### Membranous Nephropathy

We found one prospective case series publication on treatment with OFA in three patients with PLA_2_R-mediated membranous nephropathy (40).

Patient 1 was a 74-year-old man suffering from nephrotic syndrome positive for anti-PLA_2_R antibodies. After ineffective treatment with RTX, he was assigned to receive three cycles of double-filtration plasmapheresis (DFPP) followed by OFA. Despite the depletion of B cells, anti-PLA_2_R levels remained high and the nephrotic syndrome persisted leading to end-stage renal disease.

The second patient, a 69-year-old man, experienced an anaphylactic reaction after a single RTX infusion, which was associated with a transient reduction of anti-PLA_2_R antibodies. Thus, he was offered a rescue therapy with OFA and DFPP. Six days after a 100 mg OFA infusion he received three cycles of DFPP. Anti-PLA_2_R titers remained low for three months but increased again to pretreatment levels after six months. Accordingly, proteinuria persisted.

The third patient, a 80-year-old man, had very high anti-PLA_2_R titers and was treated with 100 mg OFA followed by 4 cycles of DFPP. During follow-up, anti-PLA_2_R antibody titers decreased and were undetectable at six months. Partial remission of nephrotic syndrome was observed.

The study did not report safety data or effects on QoL.

*Synopsis*: The available case series included only three patients with rather negative results. Only one of three treated patients achieved partial remission of kidney disease.

##### Multiple Sclerosis

We identified four placebo-controlled RCTs using OFA in 1136 patients with RRMS (41–43) or secondary progressive MS (43) according to the McDonald criteria. The treatment period lasted 24, 48 weeks, and 30 months, respectively. Patients were aged between 18-55 years old and had an EDSS of 0 to 5 (41) or 5.5 (42, 43).

In the study of Sorensen et al. (41), patients received two doses OFA (100 mg, 300 mg, or 700 mg) or placebo IV two weeks apart. After 24 weeks, treatment was switched and another two infusions were administered in a blinded manner. The primary endpoint was safety. There were significant reductions noted in the number of new GdE T1 lesions, total number of GdE T1 lesions, and new and/or enlarging T2 lesions (41). However, there were no significant changes found in the EDSS score.

In the MIRROR study (42), patients received OFA 3 mg, 30 mg, or 60 mg every 12 weeks subcutaneously (SC). A fourth treatment arm received OFA 60 mg SC every four weeks. The cumulative number of new GdE lesions at week 12 was the primary endpoint and was found to be reduced by 65% in patients receiving OFA (p<0.001). However, there was no significant difference concerning EDSS and relapse rates (42).

The ASCLEPIOS I and II trials were multicenter RCTs conducted concurrently and following the same study design (43). 20 mg OFA were administered SC every four weeks with loading doses on days one, seven, and 14. After one month of treatment, patients were allowed to apply the medication at home. The control group received daily teriflunomide orally. Both groups received matching placebo in order to blind the study. The primary endpoint, reduction in annualized relapse rate, was achieved in both trials. For the secondary endpoints a pooled analysis of both trials was performed, which showed a significant reduction in disability worsening at three and six months, whereas there was no significant disability improvement noted. While there was a significant reduction in GdE T1 and T2 lesions in the OFA groups, the annually brain volume loss was comparable in the teriflunomide and OFA groups.

The frequency of AEs and SAEs was comparable between OFA and placebo in all studies. There was one death in the teriflunomide group of the ACLEPIOS II trial.

QoL was not analyzed.

*Synopsis:* Based on the available data, the FDA and EMA approved subcutaneous OFA for the treatment of patients with RRMS or secondary progressive MS.

##### Rheumatoid Arthritis

Four placebo-controlled RCTs (44–47) including 852 patients investigating OFA in RA patients were included in our systematic review. The main inclusion criteria were diagnosis of RA according to the ACR criteria with a minimum disease duration of six months and a patient age of at least 18 years. In all but one trial (47), disease needed to be active. Concomitant treatment with DMARDs, another autoimmune disease, and significant comorbidity were the most important exclusion criteria.

Except in the trial by Kurrasch et al. (44) where patients received a single SC dose, IV OFA was given with a dosing interval of two weeks. IV doses ranged from 300 mg to 1000 mg. All patients were allowed to receive concomitant methotrexate and oral corticosteroids at stable dosages. In all, except the SC trial (44), non-steroidal anti-inflammatory drugs, analgesics and one inter-articular injection of corticosteroids were permitted. Premedication consisted of acetaminophen, an antihistamine, and corticosteroids. Only Kurrasch et al. did not administer corticosteroids as premedication (44).

Kurrasch et al. (44) and part A of Ostergaard et al. (45) investigated safety as primary endpoint. Part B of Ostergaard et al. (45) assessed the proportion of patients with an ACR20 improvement as primary endpoint, while the extension trial explored time to treatment withdrawal. The third RCT assessed the ACR20 response rate at week 24 (47). Secondary endpoints comprised pharmacokinetics, anti-drug antibodies, EULAR responses, DAS28 response, and B cell depletion.

Both studies with available results demonstrated significantly better outcomes for OFA-treated patients (p<0.001 for both studies) when ACR20 was assessed. Ostergaard et al. also proved superiority in ACR50 response rates and proportion of patients with EULAR good or moderate response (45). Similarly, Taylor et al. (47) reported significantly better results concerning ACR50/70 response rates, proportion of patients with good or moderate EULAR response, and change in DAS28-ESR or DAS28–C-reactive protein. The open-label study of Quattrocchi et al. was terminated early due to the study sponsor’s refocus on the investigation of SC administration and no efficacy results were available at study termination (46). Kurrasch et al. did not report markers of disease activity (44).

All four studies determined the occurrence of AEs. The incidence of AEs in OFA-treated patients ranged from 85% to 89% and in the placebo group from 55% to 62.5%. Thus, AEs occurred with a numerically but not significantly higher frequency in patients treated with OFA. SAEs occurred in 3.7%, 5%, 9.4%, 9.5%, 13%, and 20% of the OFA-treated patients compared with 0%, 0%, 3%, 5%, and 7% of the placebo treated-patients. Only one death was reported (interstitial lung disease) occurring in a patient that received 700 mg OFA.

Health-related QoL was assessed in the study of Taylor et al. (47) using scoring by FACIT-F and version 2 of the 36-Item Short Form Health Survey. For both scores significant improvements were seen in OFA-treated patients (47).

*Synopsis:* Available results show that OFA in combination with methotrexate is more effective than placebo treatment. There were no safety concerns. However, results from SC administered OFA are sparse and need further investigation.

##### Systemic Lupus Erythematosus

One study matched our inclusion criteria assessing OFA in SLE patients with refractory lupus nephritis (48). It was a case series including four patients with initial response to RTX, however, during the course of RTX treatment, patients had developed infusion reactions and were thus treated with OFA.

IV OFA was administered at different dosing regimens. All patients received prednisolone as concomitant treatment. One patient was additionally treated with cyclosporine A and another with antimalarial drugs. No primary nor secondary endpoints were defined.

The efficacy of OFA treatment was assessed using the urine albumin-to-creatinine ratio. Although it decreased in all four patients, only one reached normalization.

The only observed AE occurring in one patient one day after OFA infusion was widespread urticaria, which caused discontinuation of OFA in that patient.

The influence on their QoL was not assessed.

*Synopsis:* Available results are sparse but indicate a treatment effect in SLE patients with lupus nephritis. However, RCTs involving more patients are needed to confirm these initial findings.

#### Ublituximab

##### Multiple Sclerosis

We found one study assessing UBL in 48 patients with relapsing-remitting MS, as defined by the 2010 McDonald criteria (49). Patients were randomized to receive either placebo (12 patients) or UBL (36 patients), within six cohorts treated with different doses (450 mg or 600 mg) given over 1–4 hours of infusion. The study was unblinded on day 28 and patients in the placebo group could cross over to the corresponding treatment group.

The primary endpoint, CD19^+^ B cell depletion of at least 95%, was achieved in all patients receiving UBL. In most patients CD19^+^ B cell depletion was achieved within 24 hours after the first dose of UBL and was maintained for up to 48 weeks. No new or persisting GdE T1 lesions were observed, however, 8 patients developed one or more new GdE T2 lesions. 93% of all patients remained relapse-free, and, overall, 74% had no evidence of disease activity.

UBL was well tolerated, with infusion-related reactions representing the most common AEs. There was only one SAE observed. No deaths were reported.

*Synopsis*: UBL was well tolerated and resulted in a significant reduction of circulating CD19^+^ B cells and a reduced annualized relapse rate of MS. However, the included number of patients was too small to conclude. Moreover, future studies should compare UBL to established treatments of MS.

##### Neuromyelitis Optica Spectrum Disorder

One phase I open-label study tested UBL in patients with neuromyelitis optica spectrum disorder (NMOSD) (50). 5 patients with NMOSD and new neurological symptoms received an IV infusion of 450 mg UBL in addition to standard treatment with IV methylprednisolone.

The primary endpoint was safety. Secondary endpoints included efficacy and assessment of B cell counts. Efficacy was assessed by measuring the EDSS score at baseline, during relapse, at discharge, and at a 90-day follow-up visit. Overall, EDSS increased from 4.0 at baseline to 6.5 during relapse and remained high until discharge. However, it returned to 4.0 at the 90-day follow-up visit.

Only one patient experienced a SAE, which was leukopenia without corresponding symptoms or complications.

*Synopsis*: This small phase I study using UBL in NMOSD patients showed promising safety results. However, the currently available data on the efficacy of UBL in NMOSD are sparse and need further assessment in RCTs.

#### Veltuzumab

##### Immune Thrombocytopenia

We identified two clinical trials conducted as open-label studies without control group matching our inclusion criteria (51, 52). The study durations were 48 weeks (51) and five years (52).

91 patients were treated with VEL during either of the two trials. Patients needed to have a diagnosis of primary immune thrombocytopenia (ITP) according to the American Society of Hematology guidelines with a platelet count <30 x 10^9^ g/L on two separate occasions to enter the study. Marked or major bleeding were exclusion criteria.

VEL was either given IV (51) or SC (51, 52). All but one treatment arm, which received weekly VEL, was treated with two doses given two weeks apart. Single doses ranged from 80 to 320 mg. One study permitted the concomitant use of prednisone and danazol if given at stable doses (51), whereas a second trial only allowed concomitant prednisone (52). Before IV administration, antipyretics and antihistamines were given as premedication.

Both studies had no predefined primary endpoint. However, studies were planned to determine safety, efficacy, pharmacodynamics, pharmacokinetics, and immunogenicity.

Efficacy was assessed through objective response, corresponding to a platelet count of ≥30 x 10^9^ g/L measured twice at least one week apart with at least two-fold increase from baseline count, and complete response, corresponding to a platelet count of ≥100 x 10^9^ g/L. Of the IV treated patients 67% achieved an objective response with 33% complete responders. SC administration led to 53% and 49% objective responses and 28% and 32% complete responses in the two studies, respectively. Median time to relapse was eight months (51) and 1.3 years (52), respectively. One study also reported a bleeding reduction in all treatment groups (52).

71.4% of the IV VEL-treated patients had at least one treatment-related AE, whereas 73.5% and 78% of the SC groups had at least one AE. A total of two SAEs occurred, one in a SC treated patient (grade 3 viral gastroenteritis) and one in a patient receiving IV VEL (grade 3 hypersensitivity reaction).

Neither of the studies assessed QoL.

*Synopsis:* Available efficacy results of VEL treatment of 91 patients suffering from primary ITP seemed promising, with no unexpected safety events. However, both studies were conducted as open-label uncontrolled trials making the available data rather unreliable. Thus, blinded RCTs need to verify the results reported above.

### Risk of Bias Assessment

We assessed the quality and risk of bias of the included studies using a modified Downs and Black checklist (**Table 3**).

**Table 3:** Risk of bias.

	Reporting										External validity			Internal validity								Source of patients included					Power	Summary
	1	2	3	4	5	6	7	8	9	10	11	12	13	14	15	16	17	18	19	20	21	22	23	24	25	26	27	
**Obinutuzumab**																												
**Membranous nephropathy**																												
Klomjit et al., 2020 (26)	x	o	x	x	o	x	o	o	x	–	o	o	o	o	o	o	–	o	x	x	o	o	–	–	–	o	–	7
Furie et al., 2021 (27)	x	x	x	x	x	x	x	x	x	x	o	o	o	x	x	x	x	x	o	x	o	x	x	x	o	x	x	21
**Ocrelizumab**																												
**Multiple sclerosis**																												
Kappos et al., 2011 (28)	x	x	x	x	x	x	x	x	x	x	o	o	o	x	x	x	x	x	x	x	o	o	x	x	–	x	x	22
Hauser et al., 2017 (OPERA I trial) (29)	x	x	x	x	x	x	x	x	x	x	o	o	o	x	x	x	x	x	x	x	o	x	x	x	x	x	x	24
Montalban et al., 2017 (30)	x	x	x	x	x	x	x	x	x	x	o	o	o	x	x	x	x	x	x	x	o	o	x	x	x	x	x	23
**Rheumatoid arthritits**																												
Genovese et al., 2008 (ACTION trial) (33)	x	x	x	x	x	x	–	x	o	–	o	o	o	x	x	x	x	x	o	x	o	o	x	x	–	x	–	17
Harigai et al., 2012 (34)	x	x	x	x	x	o	–	x	x	x	o	o	o	x	o	x	x	x	x	x	o	o	x	o	–	x	–	17
Rigby et al., 2012 (STAGE trial) (35)	x	x	x	x	x	x	x	x	x	x	o	o	o	x	x	x	x	x	x	x	o	o	x	x	x	x	x	23
Stohl et al., 2012 (FILM trial) (36)	x	x	x	x	x	o	x	x	x	x	o	o	o	x	x	x	x	x	x	x	o	x	x	o	x	x	x	22
Tak et al., 2012 (SCRIPT trial) (37)	x	x	x	x	x	x	x	x	x	x	o	o	o	x	x	x	x	x	x	x	o	o	o	o	x	x	x	21
**Systemic lupus erythematosus**																												
Mysler et al., 2013 (38)	x	x	x	x	x	o	x	x	x	x	o	o	o	x	o	x	x	x	x	x	o	o	o	o	x	–	–	17
**Ofatumumab**																												
**ANCA-associated vasculitis**																												
McAdoo et al., 2016 (39)	x	–	–	x	x	x	–	x	–	–	o	x	o	–	–	x	o	o	o	x	o	o	–	–	–	o	–	9
**Multiple sclerosis**																												
Sorensen et al., 2014 (41)	x	x	x	x	x	x	–	x	x	x	o	o	o	x	o	x	x	x	x	x	o	o	x	o	x	x	–	19
Bar-Or et al., 2018 (MIRROR trial) (42)	x	x	x	x	x	x	x	x	o	x	o	o	o	x	x	x	x	x	o	x	o	o	x	x	x	x	x	21
Hauser et al., 2020 (43)	x	x	x	x	x	x	x	x	x	x	o	o	o	x	x	x	x	x	o	x	o	o	x	x	x	x	x	22
**Rheumatoid arthritis**																												
Ostergaard et al., 2010 (45)	x	x	x	x	x	x	x	x	x	x	o	o	o	x	o	x	x	x	x	x	o	o	x	o	x	x	x	21
Taylor et al., 2011 (47)	x	x	x	x	x	x	x	x	x	x	o	o	o	x	x	x	x	x	x	x	o	o	x	x	x	x	x	23
Kurrasch et al., 2013 (44)	x	x	x	x	x	o	–	x	x	–	o	o	o	x	–	x	x	o	x	x	o	o	x	–	–	x	–	15
Quattrocchi et al., 2016 (46)	x	x	x	x	x	o	–	x	x	–	o	o	o	x	x	o	o	–	o	o	o	o	x	x	x	o	–	13
Quattrocchi et al., 2016 (Extension trial) (46)	x	x	x	x	x	o	–	x	x	–	o	o	o	–	–	o	o	–	o	o	o	o	–	–	x	o	–	9
**Systemic lupus erythematosus**																												
Haarhaus et al., 2016 (48)	x	–	–	x	–	o	–	o	–	–	o	o	o	–	–	o	o	o	o	–	o	o	–	–	–	o	–	2
**Ublituximab**																												
**Multiple sclerosis**																												
Fox et al., 2021 (49)	x	x	x	x	–	x	x	–	–	x	o	o	o	o	–	o	–	o	o	x	o	o	o	o	o	o	–	8
**Neuromyelitis optica spectrum disorder**																												
Mealy et al., 2019 (50)	x	–	x	–	–	–	–	–	–	–	–	–	–	–	–	o	–	o	o	–	–	o	–	–	–	o	–	2
**Veltuzumab**																												
**Immune thrombocytopenia**																												
Liebman et al., 2013 (51)	x	x	x	x	x	x	–	x	–	–	o	o	o	–	–	x	o	o	o	x	o	o	–	–	x	o	–	11
Liebman et al., 2016 (52)	x	x	x	x	x	x	–	x	–	–	o	o	o	–	–	x	o	o	o	x	o	o	–	–	x	o	–	11

## Discussion

To provide a prompt synopsis we created a table summarizing the current state of research and clinical efficacy of OBI, OCR, OFA, UBL and VEL (**Table 4**). To address safety we also created a table highlighting the AEs reported in the studies included in this systematic review (**Table 5**). However for most of the included biologics only short-term safety data were available. Long-term safety data should be obtained in future studies testing these CD20-targeting biologics and should also assess their combination with other immunosuppressive drugs. As mentioned in the introduction, the repeated use of RTX in combination with high doses of corticosteroids and MMF has been found to increase the risk of persistent hypogammaglobulinemia (17).

**Table 4:** Summary of the evidence.

Biologic	Obinutuzumab		Ocrelizumab			Ofatumumab					Ublituximab		Veltuzumab
Disease	PLA_2_R-MN	SLE	MS	RA	SLE	AAV	MS	PLA_2_R-MN	RA	SLE	MS	NMOSD	ITP
Level I													
Level IIa													
Level IIb				*	*								
Level IIIa													
Level IIIb													
Level IV													
Too little information													

Level I  Approved by the EMA and/or FDA.

Level IIa  Multicentric double-blind RCTs proving a significant superiority over standard-of-care treatment.

Level IIb  Multicentric double-blind RCTs proving a significant superiority over placebo.

Level IIIa Clinical study, not fulfilling the above-mentioned criteria, but proving a superiority over standard-of-care treatment.

Level IIIb Clinical study, not fulfilling the above-mentioned criteria, but proving a superiority over placebo.

Level IV  Case series or open-label trials without control group with positive results.

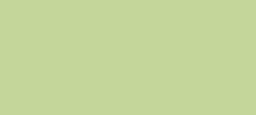
 Achieved

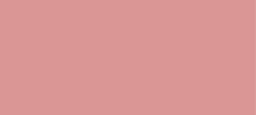
 Failed

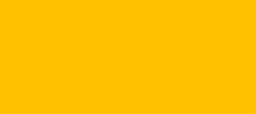
 Mixed result

*Increased risk of serious infections.

AAV, anti-neutrophil cytoplasmic antibody (ANCA)-associated vasculitis; EMA, European Medicines Agency; FDA, U.S. Food and Drug Administration; ITP, immune thrombocytopenia; MS, multiple sclerosis; PLA_2_R-MN, phospholipase A_2_ receptor-associated membranous nephropathy; RA, rheumatoid arthritis; RCT, randomized controlled trial; SLE, systemic lupus erythematosus.

**Table 5:** Adverse events.

Obinutuzumab			
Organ systems affected	Adverse event(s)		Refs.
**Systemic**	**a) Immediate-type adverse reactions**	Infusion reaction	(27)
	**b) Infection**	Urinary tract infection, bronchitis, herpes zoster, upper respiratory tract infection, influenza, gastroenteritis	(26, 27)
	**c) Neoplasm**	None reported. Further studies in patients with immune-mediated diseases are needed to determine the frequency.	
**Cardiovascular**	Hypertension, peripheral edema		(27)
**Gastrointestinal and hepatic**	Abdominal pain, nausea, diarrhea		(27)
**Hematologic events**	Anemia, neutropenia		(27)
**Musculoskeletal**	Arthralgia		(27)
**Nervous system (including eyes)**	Headache, conjunctivitis, insomnia		(29, 30, 33–36, 38)
**Renal**	None reported. Further studies in patients with immune-mediated diseases are needed to determine the frequency.		
**Upper and lower airways**	Nasopharyngitis, pharyngitis, sinusitis, cough		(27)
**Urogenital**	Frequent urination		(27)
**Skin**	None reported. Further studies in patients with immune-mediated diseases are needed to determine the frequency.		
**Ocrelizumab**			
**Organ systems affected**	**Adverse event(s)**		**Refs.**
**Systemic**	**d) Immediate-type adverse reactions**	Infusion reaction	(28–38)
	**e) Infection**	Upper respiratory tract infection, oral herpes, typhoid fever, urinary tract infection, urosepsis, bacterial arthritis, sepsis, septic shock	(28–38)
	**f) Neoplasm**	Breast cancer, cervix cancer, endometrial cancer, ovarian cancer, bladder cancer, renal-cell carcinoma, basal cell carcinoma, squamous cell carcinoma, malignant melanoma, laryngeal cancer, lung cancer, adenocarcinoma of colon, esophageal adenocarcinoma, pancreatic carcinoma, lymphoma, malignant fibrous histiocytoma, papillary thyroid cancer	(29, 30, 32–37)
**Cardiovascular**	Chest pain, hypertension, hypotension, pallor, bradycardia, tachycardia, palpitations, ventricular extrasystole, myocardial infarction		(31, 35–38)
**Gastrointestinal and hepatic**	Nausea, dysphagia, dyspepsia, odynophagia, oral pain, diarrhea, constipation, esophagitis, elevated liver enzyme values, appendicitis		(31, 33, 35–38)
**Hematologic events**	Neutropenia, disseminated intravascular coagulopathy, hypogammaglobulinemia		(28, 31–34, 36–38)
**Musculoskeletal**	Back pain, pain in extremity, arthralgia, myalgia		(30–32, 35–37)
**Nervous system (including eyes)**	Headache, migraine, conjunctivitis, fatigue, sensory disturbance, tremor, somnolence, vertigo, depression, stroke, cerebral hemorrhage, suicide		(29–36, 38)
**Renal**	Acute renal failure		(38)
**Upper and lower airways**	Nasopharyngitis, nasal congestion, throat irritation, dyspnea, pharyngeal swelling, oropharyngeal edema, bronchitis, bronchospasm, pneumonia, pulmonary embolism		(29–32, 34–38)
**Urogenital**	Epididymitis, cystitis		(31, 34)
**Skin**	Pruritus, rash, flushing, urticaria, angioedema, erythema, cellulitis		(29, 31, 34–38)
**Ofatumumab**			
**Organ systems affected**	**Adverse event(s)**		**Refs.**
**Systemic**	**a) Immediate-type adverse reactions**	Infusion reaction	(39, 41–48)
	**b) Infection**	Upper respiratory tract infection, urinary tract infection, genital infections, tooth infection, skin infections, sepsis	(39, 41–47)
	**c) Neoplasm**	Breast cancer, ovarian cancer, malignant melanoma, basal cell carcinoma, lymphoma, gingival carcinoma	(42–46)
**Cardiovascular**	Tachycardia, bradycardia, palpitations, hypertension, hypotension, atrial fibrillation, atrioventricular block, cardiac ischemia, pericardial effusion, left ventricular hypertrophy		(43–45, 47)
**Gastrointestinal and hepatic**	Nausea, vomiting, abdominal pain, dysphagia, dyspepsia, stomatitis, duodenal ulcer, diarrhea, constipation, gastroenteritis, cholelithiasis, diverticulitis, pancreatic necrosis, elevated liver enzyme values, appendicitis		(42–47)
**Hematologic events**	Anemia, leukopenia, neutropenia, lymphopenia, thrombocytosis, eosinophilia		(39, 43, 46)
**Musculoskeletal**	Back pain, pain in extremity, synovitis, bursitis, arthritis, myalgia		(41, 42, 45, 47)
**Nervous system (including eyes)**	Headache, fatigue, periorbital edema, vertigo, tinnitus, ear pain, hypoacusis, deafness, hypothyroidism, eye disorder (blurred vision, eye pain, diplopia, dry eye, blepharospasm, conjunctivitis, cataract, chalazion), paresthesia, migraine, syncope, tremor, somnolence, restless legs syndrome, amnesia, myasthenia gravis, depression, anxiety, insomnia, suicide attempt		(41–47)
**Renal**	Nephrolithiasis, pollakiuria, hematuria, leukocyturia, proteinuria		(43)
**Upper and lower airways**	Nasopharyngitis, throat irritation, laryngitis, sinusitis, bronchitis, cough, bronchospasm, pneumonia, interstitial lung disease, pulmonary embolism		(39, 41–47)
**Urogenital**	Endometritis, urinary incontinence, menorrhagia, dysmenorrhea, cervical dysplasia, erectile dysfunction, balanoposthitis		(46)
**Skin**	Pruritus, rash, flushing, erythema, urticaria, angioedema, alopecia		(41, 42, 45–47)
**Ublituximab**			
**Organ systems affected**	**Adverse event(s)**		**Refs.**
**Systemic**	**a) Immediate-type adverse reactions**	Infusion reaction	(49)
	**b) Infection**	Upper respiratory tract infection, influenza, fungal infection	(49)
	**c) Neoplasm**	None reported. Further studies in patients with immune-mediated diseases are needed to determine the frequency.	
**Cardiovascular**	None reported. Further studies in patients with immune-mediated diseases are needed to determine the frequency.		
**Gastrointestinal and hepatic**	Nausea, diarrhea, constipation, upper abdominal pain, vomiting		(49)
**Hematologic events**	Leukopenia		(50)
**Musculoskeletal**	Arthralgia, back pain		
**Nervous system (including eyes)**	Dizziness, fatigue, headache, contusion, depression, blurred vision		(49, 50)
**Renal**	None reported. Further studies in patients with immune-mediated diseases are needed to determine the frequency.		
**Upper and lower airways**	Cough, nasopharyngitis, sinusitis		(49)
**Urogenital**	None reported. Further studies in patients with immune-mediated diseases are needed to determine the frequency.		
**Skin**	Rash		(49)
**Veltuzumab**			
**Organ systems affected**	**Adverse event(s)**		**Refs.**
**Systemic**	**d) Immediate-type adverse reactions**	Infusion reaction	(51, 52)
	**e) Infection**	Upper respiratory tract infection, urinary tract infection	(51, 52)
	**f) Neoplasm**	None reported	
**Cardiovascular**	Atrial fibrillation, palpitations		(51, 52)
**Gastrointestinal and hepatic**	Nausea, vomiting, abdominal pain, abdominal bloating, gastroenteritis, dyspepsia, elevated liver enzyme values		(51, 52)
**Hematologic events**	Bleeding, neutropenia, lymphopenia, thrombocytopenia		(51, 52)
**Musculoskeletal**	Pain in extremity, myalgia, back pain, chest pain		(51, 52)
**Nervous system (including eyes)**	Headache, fatigue, peripheral neuropathy		(51, 52)
**Renal**	Elevated creatinine values, chronic renal failure		(51)
**Upper and lower airways**	Nasopharyngitis, sinusitis, throat irritation		(51, 52)
**Urogenital**	Increased thirst and urination		(52)
**Skin**	Pruritus, burning, erythema, swelling, edema, bruising, cellulitis		(51, 52)

OBI allowed an improvement of nephrotic syndrome-grade proteinuria and of serum albumin concentrations in three patients with PLA_2_R-associated membranous nephropathy refractory to treatment with RTX. Based on these promising results, RCTs using OBI in PLA_2_R-associated membranous nephropathy are warranted. Moreover, OBI was tested in SLE patients with active lupus nephritis and led to a significantly improved overall response in comparison to placebo.

OCR achieved a significant reduction of the annualized relapse rate in patients with RRMS as well as a significantly lower disease progression in PPMS patients, thus the EMA and FDA approved its administration in patients with PPMS or RRMS. OCR was further used in patients with RA leading to significant improvement of ACR rates. However, rates of serious infections were elevated with use of OCR. These safety concerns were also raised in SLE patients treated with OCR, leading to early termination of the only available RCT. Thus, a close post-marketing monitoring of MS patients treated with OCR is warranted.

For the use of OFA in patients with AAV, PLA_2_R-associated membranous nephropathy, and SLE, there were only case series available. The studies in AAV and SLE showed promising results, whereas the data in patients with membranous nephropathy were rather negative. Furthermore, eight RCTs assessed the use of OFA in patients with either RRMS, secondary progressive MS, or RA where treatment with OFA resulted in a significant clinical improvement with no increased safety concerns. Thus, OFA was approved by the FDA and EMA for use in RRMS and in secondary progressive MS.

UBL was tested in a placebo-controlled RCT with MS patients and showed an improvement in frequency of T1 lesions and volume of T2 lesions. A phase I trial in patients with NMOSD showed promising safety data, whereas the trial was too small to conclude on efficacy.

VEL has only been tested in patients with ITP and showed a positive influence on platelet counts and bleeding complication in the available open-label trials.

### Limitations

This is the first systematic review on the safety and efficacy of OBI, OCR, OFA, UBL and VEL in a number of immune-mediated diseases. We have used standardized systematic overview techniques, which have helped to minimize the risk of bias. Furthermore, we assessed the quality and bias of each study using a modified version of the Downs and Black checklist.

Nonetheless, our systematic review has several limitations. Firstly, we included studies with different outcome measures, inclusion criteria, concomitant treatment, premedication, control groups, and study duration, making a direct comparison difficult. Since we also considered certain case series and open-label trials, the reported results may be influenced by chance and may in consequence not be as reliable as those found by a double-blind RCTs involving more patients. Furthermore, we did not assess for risk of bias across the studies. However, we aimed to minimize the risk by double-checking the presented data as well as the inclusion of trials.

### Conclusions

OBI appeared to be beneficial in three patients with PLA_2_R-associated membranous nephropathy who were refractory to treatment with RTX. OBI was also tested in SLE patients with active lupus nephritis with mixed results. OCR was approved by the EMA and FDA for treatment of patients with RRMS or PPMS. Furthermore, OCR showed promising or mixed results in patients with RA or SLE, respectively, however, in these trials, OCR was associated with an increased rate of serious infections. OFA was approved by the EMA and FDA for its use in RRMS. Moreover, OFA was tested in patients with AAV, RA, and SLE and resulted in disease improvement. Conversely, OFA showed mixed results in patients with PLA_2_R-associated membranous nephropathy. UBL was tested in MS and in NMOSD, revealing promising results, although the numbers of treated patients were small. VEL was tried in patients with ITP in open-label designed studies and appeared to be effective.

## Data Availability Statement

The original contributions presented in the study are included in the article/**Supplementary Material**. Further inquiries can be directed to the corresponding author.

## Author Contributions

Conception and design of the work: CK and OB. Data collection: CK and BW. Data analysis and interpretation: CK and BW. Drafting the article: CK, CC, and OB. Critical revision of the article: CK, BW, CC, and OB. Final approval of the version to be published: CK, BW, CC, and OB.

## Conflict of Interest

The authors declare that the research was conducted in the absence of any commercial or financial relationships that could be construed as a potential conflict of interest.

## Publisher’s Note

All claims expressed in this article are solely those of the authors and do not necessarily represent those of their affiliated organizations, or those of the publisher, the editors and the reviewers. Any product that may be evaluated in this article, or claim that may be made by its manufacturer, is not guaranteed or endorsed by the publisher.

## Abbreviations

AAV, ANCA-associated vasculitis; ACR, American College of Rheumatology; AE, adverse event; ANCA, anti-neutrophil cytoplasmic antibody; anti-PLA_2_R, anti-phospholipase A_2_ receptor; CD, cluster of differentiation; CDC, complement-dependent cytotoxicity; DAS28, 28-joint disease activity score; DAS28-ESR, DAS28–erythrocyte sedimentation rate; DFPP, double-filtration plasmapheresis; DMARD, disease-modifying antirheumatic drug; EDSS, expanded disability status scale; eGFR, estimated glomerular filtration rate; EMA, European Medicines Agency; ESR, erythrocyte sedimentation rate; EULAR, European League Against Rheumatism; FDA, U.S. Food and Drug Administration; Fc, fragment crystallizable; GdE, gadolinium-enhancing; IRR, infusion-related reaction; ITP, immune thrombocytopenia; IV, intravenous(ly); mAb, monoclonal antibody; MMF, mycophenolate mofetil; MS, multiple sclerosis; NMOSD, neuromyelitis optica spectrum disorder; OBI, obinutuzumab; OCR, ocrelizumab; OFA, ofatumumab; PGA, patient global assessment; PLA_2_R, phospholipase A_2_ receptor; PPMS, primary progressive multiple sclerosis; QoL, quality of life; RA, rheumatoid arthritis; RCT, randomized controlled trial; RRMS, relapsing-remitting multiple sclerosis; RTX, rituximab; SC, subcutaneous(ly); SAE, serious adverse event; SLE, systemic lupus erythematosus; TADAI, total adjusted disease activity index; UBL, ublituximab; VEL, veltuzumab.
