# Supplementary material for: Systematic Review of Safety and Efficacy of Second- and Third-Generation CD20-Targeting Biologics in Treating Immune-Mediated Disorders
Source: Front Immunol. 2022 Feb 2;12:788830. doi: 10.3389/fimmu.2021.788830 (PMC8847774; doi:10.3389/fimmu.2021.788830)
Supplement: Supplementary file 2 [file Table_2.docx]

| **Table S2: Main characteristics of studies included in the systematic review.** | | | | | | | | | | | | | | |
| --- | --- | --- | --- | --- | --- | --- | --- | --- | --- | --- | --- | --- | --- | --- |
| **Obinutuzumab (OBI)** | | | | | | | | | | | | | | |
| **Membranous nephropathy** | | | | | | | | | | | | | | |
| **Source** | **Patients treated** | **Com­pleting verum** | **Com­pleting control** | **OBI** | **Route** | **Control** | **Concomi­tant therapy** | **Inclusion criteria** | **Primary endpoint (1°)** | **Secondary endpoint (2°)** | **# Verum reaching 1°** | **# Verum reaching 2°** | **AEs** | **SAEs** |
| Klomjit et al., 2020 | 3 | 3 | No control | Patient 1: 100mg on day 1 and 900mg on day 2.  Patient 2: 100mg on day 1 and 900mg on day 2 and 1g on day 8.  Patient 3: 100mg on day 1, 900mg on day 2 and 1g on day 15. | IV | No control | Patient 1:  Premed: IV MEP 40mg, 25mg diphenhydramine, 650mg acetaminophen.  Patient 2: Premed with steroids and AH.  Patient 3: unknown. | PLA2R associated membranous nephropathy not responding to RTX treatment | No defined primary endpoint | No defined secondary endpoints | Patient 1: reduction of proteinuria from 6.8g/d to 1.1g/d after 18 months.  Patient 2: reduction of proteinuria from 20.4g/d to 6.8g/d after 9 months.  Patient 3: reduction of proteinuria from 19.5g/d to 1.5g/d after 24 months. | - | Patient 1 and 2: no side effects. Patient 3: localized herpes zoster 1 month after OBI. | Patient 1, 2 and 3: no SAES. |
| Furie et al., 2021 | 125 | 57 (ITT 64) | 46 (ITT 62) | 1000mg on day 1 and weeks 2, 24, and 26. | IV | Placebo | All patients received MMF at a dose of 2-2.5g/d as well as corticosteroids. Furthermore, a treatment with antimalarial medications, ACE inhibitors or angiotensin receptor blocker, calcium and vitamin D was recommended.  Premed: 80mg IV MEP, | Diagnosis of SLE according to the ACR 1997 criteria, age 18-75 years, kidney biopsy with class III or IV active or active/chronic lupus nephritis during the last 6 months, eGFR ≥30ml/min/1.73m2 | Proportion of patients with complete renal response (CRR) at week 52. | Proportion of patients achieving partial renal response or overall renal response (ORR), changes in C3, C4 and anti-dsDNA, time to CRR and ORR | 22/63 (35%) patients achieved CRR vs 14/62 (23%) in the placebo group (p=0.115) | Significant change from baseline concerning C3 (p<0.001) and C4 (p<0.001) levels. Significantly more patients achieved an ORR (p0.025). | 58/64 patients with at least one AE (91%).  PBO: 54/61 patients (89%). | 16/16 Patients (25%) with at least one SAE. 5 deaths, 1 in OBI group (gastrointestinal perforation), 4 in the PBO group (gastrointestinal hemorrhage, refractory SLE, PML, respiratory infection). |

| **Ocrelizumab (OCR)** | | | | | | | | | | | | | | |
| --- | --- | --- | --- | --- | --- | --- | --- | --- | --- | --- | --- | --- | --- | --- |
| **Multiple sclerosis** | | | | | | | | | | | | | | |
| **Source** | **Patients treated** | **Com­pleting verum** | **Com­pleting control** | **OCR** | **Route** | **Control** | **Concomi­tant therapy** | **Inclusion criteria** | **Primary endpoint (1°)** | **Secondary endpoint (2°)** | **# Verum reaching 1°** | **# Verum reaching 2°** | **AEs** | **SAEs** |
| Kappos et al., 2011 | 220 | 51  (ITT 55) | 54  (ITT 54) | 300 mg on days 1 and 15 of the first cycle, 600 mg on day 1 of the second cycle, third, and fourth cycle (week 24, 48, 72) | IV | Placebo on days 1 and 15, with the option of OCR 300 mg on days 1 and 15 of the second, third, and fourth cycle (week 24, 48, 72) | Premed: IV MEP 100 mg with the option of adding an oral analgesic or antipyretic and an oral AH | Diagnosis of RRMS, 2 or more docu­mented relapses within 3 years before screening, at least 1 within the past year, EDSS of 1-6 points, evidence of previous MS inflammatory disease activity with 6 or more T2 lesions per MRI, or 2 relapses in the year before screening | Total number of GdE T1 lesions at weeks 12, 16, 20, and 24 | Annualized protocol-defined relapse-rate, proportion of relapse-free patients, total of GdE T1 lesions, total number of new GdE T1 lesions, change in total volume of T2 lesions from baseline to week 24, safety and tolerability | Mean number of GdE T1 lesions over weeks 12, 16, 20, and 24: 0.6 (p<0.0001 vs placebo and vs IFN-β-1a) | Annua­lized relapse rate by week 24: 0.13 (p=0.0005 vs placebo and p=0.03 vs IFN-β-1a) | 34 patients (62%) | 2 patients (4%) |
|  |  | 48  (ITT 55) |  | 1000 mg on days 1 and 15, 1000 mg on day 1 of the second, third, and fourth cycle (week 24, 48, 72) | IV |  |  |  | Total number of GdE T1 lesions at weeks 12, 16, 20, and 24 |  | Mean number of GdE T1 lesions over weeks 12, 16, 20, and 24: 0.2 (p<0.0001 vs placebo and vs IFN-β-1a) | Annua­lized relapse rate by week 24: 0.17 (p=0.0014 vs placebo and p=0.09 vs IFN-β-1a) | 36 patients (66%) |  |
|  |  | 51  (ITT 54) |  | IFN-β-1a 30 µg weekly until week 24, with the option of OCR 300 mg on days 1 and 15 of the second, third, and fourth cycle (week 24, 48, 72) | IM |  |  |  | Total number of GdE T1 lesions at weeks 12, 16, 20, and 24 |  | Mean number of GdE T1 lesions over weeks 12, 16, 20, and 24: 6.9 | Annua­lized relapse rate: 0.36 (p=0.07 vs placebo) | 30 patients (56%) |  |
| Hauser et al., 2017 (OPERA I) | 821 | 366  (ITT 410) | 240  (ITT 411) | 600 mg every 24 weeks. The first dose was administered as two doses of 300 mg given on days 1 and 15 | IV | IFN-β-1a 44 ug 3 times weekly | Premed: 100 mg IV MEP. Analgesics, antipyretics, and AHs were recommen­ded as well | Diagnosis of MS according to the 2010 McDonald criteria, age 18-55, EDSS of 0-5.5, ≥ 2 documen­ted clinical relapses within previous 2 years or one relapse within the last year, MRI abnormali­ties consistent with MS. Exclusion criteria: neurologic worsening within the last 30 days, primary progressive MS, previous treatment with B cell-targeted therapy or other immuno­suppressive medication as defined in the protocol, disease duration >10 years | Annualized relapse rate at week 96 | Proportion of patients with disability progression, total mean number of GdE lesions on T1-weighted MRI scans, total number of new or newly enlarged hyperintense lesions on T2-weighted MRI scans, disability improve­ment, total number of new hypointense lesions on T1-weighted MRI scans, change in MS functional composite score, percentage change in brain volume, change in physical component summary score of 36-item short form health survey, proportion of patients with an EDSS score of ≥2 with no evidence of disease activity | 0.16 (p<0.001) | Total mean number of GdE lesions on T1 MRI: 0.02 (p<0.001). Total mean number of new or newly enlarged hyperintense T2 lesions in MRI: 0.32 (p<0.001). Total mean number of new hypointense T1 MRI lesions: 0.42 (p<0.001) | 327 patients (80.1%) | 7.8%.  1 death (suicide) |
| Hauser et al., 2017 (OPERA II) | 835 | 360  (ITT 417) | 320  (ITT 418) | 600 mg every 24 weeks. The first dose was administered as two doses of 300 mg given on days 1 and 15 | IV | IFN-β-1a 44 ug 3 times weekly | Premed: 100 mg IV MEP. Analgesics, antipyretics, and AHs were recommended as well |  | Annualized relapse rate at week 96 |  | 0.16 (p<0.001) | Total mean number of GdE lesions on T1 MRI: 0.02 (p<0.001). Total mean number of new or newly enlarged hyperin­tense T2 lesions in MRI: 0.33 (p<0.001). Total mean number of new hypoin­tense T1 MRI lesions: 0.45 (p<0.001) | 360 patients (86.3%) | 9.6% with an SAE.  1 death (mechanical ileus) |
| Hauser et al., 2020 (Open-label extension trial of OPERA I and II) | 1325 | 623 (ITT 702) from OCR group. 551 (ITT 623) from IFN-β group. A total of 1174 patients completed 3 years | No control | 600 mg every 24 weeks. First dose was administered in two doses of 300 mg given on days 1 and 15 | IV | No control | Premed: 100 mg IV MEP. Analgesics, antipyretics, and AHs were recommended as well | Diagnosis of RRMS according to the 2010 revised McDonald criteria, age 18-55 years, and EDSS of 0-5.5. Patients had to use at least 2 contraceptive methods. Exclusion criteria: life-threatening IRR, clinically significant laboratory anomaly, active infection, neutropenia, reduction of CD4^+^ cell count or serum IgG levels during the previous double-blind period | Annualized relapse rate | Disability progression / improvement, mean change in EDSS score, total number of T2 and T1 GdE lesions on MRI, and brain volume change | Previous OCR: low annualized relapse rate (0.14, 0.13, 0.10, 0.08, and 0.07 during year 1 to 5 respectively).  Previous IFN-group: significant reduction in annualized relapse rate (0.20 in year 2 to 0.10 in year 3, p<0.001), which was maintained through to year 5. During years 3-5 there was no significant difference in the annualized relapse rate between the 2 groups | Clinical disease progression and mean change in EDSS was significantly lower in patients previously randomized to the OCR-group throughout the 5 years. Clinical disease improvement was numerically higher in patients receiving OCR in double-blind phase. There was no difference in MRI lesions during years 3 to 5 between the two groups. However, brain atrophy was significantly lower in the OCR-group (p<0.01) | 220 AEs per 100 patient years | 6.1 SAEs per 100 patient years |
| Montalban et al., 2017 | 732 | 402  (ITT 488) | 174  (ITT 244) | 600 mg every 24 weeks (administered as two 300 mg-infusions 14 days apart) | IV | Placebo every 24 weeks | Premed: 100 mg IV MEP. Analgesics, antipyretics, and AHs were recommended as well | Diagnosis of PPMS according to the 2005 revised McDonald criteria, age of 18-55 years, EDSS of 3.0-6.5, score ≥ 2 on the pyramidal functions component of the Functional Systems Scale, disease duration < 15 years in patients with an EDSS of ≤5 and a documented history or presence of an elevated IgG index or at least one IgG oligoclonal band in the cerebro­spinal fluid. Exclusion criteria: history of RRMS, secondary progressive MS or progressive-relapsing MS, contrain­dications for MRI, contrain­dication or unaccep­table side effects from oral or IV CS and previous treatment with B cell-targeted therapies or other immunosuppressive medication as defined in the protocol | Percentage of patients with disability progression at week 12 (increase in the EDSS of at least 1.0 with a baseline score of at maximum 5.5 or an increase of 0.5 with a baseline score >5.5) | Percentage of patients with disability progression at week 24, change in performance on the timed 25-foot-walk, change in total volume of brain lesions on T2 weighted MRI, change in brain volume from week 24 to 120, change in physical component summary score of 36-item short form health survey version 2.0 | No information | Total volume of hyperintense T2 lesions: 3.4 (p<0.001). Change in brain volume:  -0.90 (p=0.02) | 95.1% of the patients with at least one AE | 22.2% with an SAE.  1 death (road-traffic accident) |
| Hartung et al., 2020 (ENSEMBLE PLUS substudy) | 745 | 372 | 373 | OCR 600mg every 24 weeks over a duration of 2 hours | IV | OCR 600mg every 24 weeks over a duration of 3.5 hours | AH and IV-MEP. | Diagnosis of RRMS according to the 2010 McDonald criteria, disease duration ≤3 years, ≥1 relapses/signs of MRI activity during the past 12 months, EDSS of 0-3.5. Exclusion criteria: previous serious IRR related to OCR. | Proportion of patients with IRR during the infusion and within the first 24 hours after. | Severity of IRR, IRR leading to treatment discontinuation, overall IRR, safety. | 107/372 (28.8%) versus 99/373 (26.5%) in the conventional treatment group. | IRR after 2°dose: 36/134 (26.9%) versus 26/140 (18.6%) in de conventional treatment group. IRR after 3^rd^ dose: 4/18 (22.2%) versus 1/18 (5.6%) in the conventional treatment arm. 3 severe IRR I in the shorter infusion group: headache, oropharyngeal pain/swelling, and fatigue. 1 SAE in the conventional infusion group: laryngeal inflammation. | 54.8% of patients in the shorter infusion group and 58.8% of patients in the conventional infusion group experienced an AE. | 4 SAE in the conventional and 5 in the shorter infusion group. |
| **Rheumatoid arthritis (RA)** | | | | | | | | | | | | | | |
| **Source** | **Patients treated** | **Com­pleting verum** | **Com­pleting control** | **OCR** | **Route** | **Control** | **Concomi­tant therapy** | **Inclusion criteria** | **Primary endpoint (1°)** | **Secondary endpoint (2°)** | **# Verum reaching 1°** | **# Verum reaching 2°** | **AEs** | **SAEs** |
| Genovese et al., 2008 (ACTION trial) | 237 | 32  (ITT 36) | 35  (ITT 41) | 10 mg on days 1 and 15 | IV | Placebo | All patients received concomitant MTX, starting at least 12 weeks prior. All other DMARDs had to be withdrawn. Stable doses of oral CS (≤10 mg/d) and NSAIDs were permitted. Premed: oral AH and paracetamol | Diagnosis of active RA according to the ACR criteria, inadequate response to therapy with ≤6 conventional or biologic DMARDs (including MTX at a dosage of 10-25 mg/week for ≥12 weeks) | Safety (AE and SAE) | Proportion of patients with an ACR20/50/ 70 response at week 24, DAS28 at week 24, DAS28 remission response and EULAR response categorized as good | 35 patients with an AE | No informa­tion | See primary endpoint | 6 patients (14.6%) with at least one SAE. No deaths |
|  |  | 37  (ITT 40) |  | 50 mg on days 1 and 15 | IV |  |  |  | Safety (AE and SAE) | Proportion of patients with an ACR20/50/70 response at week 24, DAS28 at week 24, DAS28 remission response and EULAR response categorized as good | 38 patients with an AE | No informa­tion | See primary endpoint |  |
|  |  | 36  (ITT 40) |  | 200 mg on days 1 and 15 | IV |  |  |  | Safety (AE and SAE) | Proportion of patients with an ACR20/50/ 70 response at week 24, DAS28 at week 24, DAS28 remission response and EULAR response categorized as good | 40 patients with an AE | No informa­tion | See primary endpoint |  |
|  |  | 36  (ITT 40) |  | 500 mg on days 1 and 15 | IV |  |  |  | Safety (AE and SAE) | Proportion of patients with an ACR20/50/ 70 response at week 24, DAS28 at week 24, DAS28 remission response and EULAR response categorized as good | 40 patients with an AE | No information | See primary endpoint |  |
|  |  | 36  (ITT 40) |  | 1000 mg on days 1 and 15 | IV |  |  |  | Safety (AE and SAE) |  | 38 patients with an AE | No information | See primary endpoint |  |
| Harigai et al., 2012 | 151 | 29  (ITT 39) | 31  (ITT 37) | 50 mg on days 1 and 15 | IV | Placebo | All patients received concomitant MTX. All other DMARDs had to be withdrawn. Stable doses of oral CS (≤10 mg/d) and NSAIDs were permitted. Rescue medications (MTX increase to 8 mg/week, nonbiologi­cal DMARD, increase of oral CS, IA CS, IA hyaluronic acid preparation, 1 biological DMARD were allowed from week 8. Premed: MEP 100 mg IV with the option of adding oral AH and paracetamol | Diagnosis of active RA according to the ACR criteria, inadequate response to MTX given for at least 12 weeks | Proportion of patients with an ACR20 response at week 24 | Proportion of patients with an ACR50/70 response, reduction in DAS28-ESR and EULAR response rates at week 24 | No information | No informa­tion | 31 patients (79.5%) with at least one AE | 3 patients (8.1%) with at least one SAE. 1 death: acute respira­tory failure |
|  |  | 28  (ITT 39) |  | 200 mg on days 1 and 15 | IV |  |  |  | Proportion of patients with an ACR20 response at week 24 | Proportion of patients with an ACR50/70 response, reduction in DAS28-ESR and EULAR response rates at week 24 | No information | No informa­tion | 31 patients (79.5%) with at least one AE |  |
|  |  | 30  (ITT 36) |  | 500 mg on days 1 and 15 | IV |  |  |  | Proportion of patients with an ACR20 response at week 24 | Proportion of patients with an ACR50/70 response, reduction in DAS28-ESR and EULAR response rates at week 24 | No information | No informa­tion | 22 patients (61.1%) with at least one AE |  |
| Rigby et al., 2012 (STAGE) | 1006 | 323  (ITT 343) | 285  (ITT 320) | 200 mg on days 1 and 15 and weeks 24 and 26 | IV | Placebo | All patients received concomitant MTX. All other DMARDS had to be withdrawn. Rescue medication was allowed. Premed: MEP 100 mg IV with optional paracetamol 1 g and AH | Diagnosis of active RA according to the ACR criteria, disease duration ≥3 months, inadequate response to MTX given for at least 12 weeks | Proportion of patients with an ACR20 response at weeks 24 and 48 | Change from baseline in the SHS and ACR50/70 responses, proportion of patients achieving remission, EULAR response, change in HAQ-DI score | No information | Higher propor­tion of patients achieved an ACR50 or 70 response at weeks 24 and 48. More patients achieved remission according to DAS28-ESR criteria at week 48. Greater reduction in joint damage (SHS) at weeks 24 and 48 | 282 patients (82.2%) with at least one AE. | 37 patients (11.6%) with at least one SAE. 1 death: mesen­teric vasculitis |
|  |  | 324  (ITT 343) |  | 500 mg on days 1 and 15 and weeks 24 and 26 | IV |  |  |  | Proportion of patients with an ACR20 response at weeks 24 and 49 | Change from baseline in the SHS and ACR50/70 responses, proportion of patients achieving remission, EULAR response, change in HAQ-DI score | No information |  | 287 patients (83.7%) with at least one AE |  |
| Stohl et al., 2012 (FILM) | 605 | 180  (ITT 196) | 183  (ITT 207) | 200 mg on days 1 and 15 and weeks 24/26, 52/54 and 76/78 | IV | Placebo | All patients received concomitant MTX. All other DMARDs had to be withdrawn. Rescue medication was allowed. Premed: MEP 100 mg IV with the option of adding paracetamol 1 g and AH. Rescue medication allowed | Diagnosis of active moderate-to-severe RA according to the ACR criteria, disease duration ≥3 months but <5 years. | Change from baseline in mTSS at week 104 (later changed to week 52) | Proportion of patients without radiographic progression and the proportion of patients achieving ACR20/50/ 70 responses at week 52. Furthermore HAQ-DI improvement and DAS28-ESR | Significant change of mTSS (p=0.001): change from baseline 0.66 | Significantly higher propor­tion of patients achieved an ACR20/ 50/70 response and a DAS28-ESR remission at week 52 | 171 patients (87.2%) with at least one AE | 21 patients (10.1%) with at least one SAE. 2 deaths: acute myocar­dial infarction and con­gestive heart failure |
|  |  | 185  (ITT 202) |  | 500 mg on days 1 and 15 and weeks 24/26, 52/54 and 76/78 | IV |  |  |  |  |  | Significant change of mTSS (p=0.0033): change from baseline 0.27 |  | 167 patients (82.7%) with at least one AE |  |
| Tak et al., 2012 (SCRIPT) | 836 | 248  (ITT 277) | 242  (ITT 277) | 200 mg on days 1 and 15 and weeks 24 and 26 | IV | Placebo | All patients received concomitant MTX or LEF. Other DMARDS were allowed if they were given for at least 12 weeks with a stable dose during the last 4 weeks, except for biological DMARDs (except TNF inhibitors), which had to be stopped. Stable doses of CS ≤10 mg/d were permitted. Premed: MEP 100 mg IV with optional paracetamol 1 g and AH | Diagnosis of active RA according to the ACR criteria, disease duration ≥ 3 months, inadequate response to previous or current treatment with at least 1 TNF inhibitor | Proportion of patients with response according to the ACR20 at weeks 24 and 48 | Change from baseline in mTSS at 48 weeks, proportion of patients with an ACR50 or ACR70 response at weeks 24 and 48, HAQ-DI at weeks 24 and 48 and the proportion of patients achieving remission according to DAS28 | No information | No informa­tion | 232 patients (83.3%) with at least one AE | 32 patients (11.6%) with at least one SAE. 1 death: fatal myocar­dial infarction |
|  |  | 261 (ITT 282) |  | 500 mg on days 1 and 15 and weeks 24 and 26 | IV | Placebo |  |  | Proportion of patients with response according to the ACR20 at weeks 24 and 48 | Change from baseline in mTSS and the ACR50/70 responses | No information | No information | 238 patients (84.4%) with at least one AE |  |
| **Systemic lupus erythematosus (SLE)** | | | | | | | | | | | | | | |
| **Source** | **Patients treated** | **Com­pleting verum** | **Com­pleting control** | **OCR** | **Route** | **Control** | **Concomi­tant therapy** | **Inclusion criteria** | **Primary endpoint (1°)** | **Secondary endpoint (2°)** | **# Verum reaching 1°** | **# Verum reaching 2°** | **AEs** | **SAEs** |
| Mysler et al., 2013 | 378 | 47  (ITT 126) | 45  (ITT 125) | 400 mg on days 1 and 15, followed by a single infusion at week 16 and every 16 weeks thereafter | IV | Placebo | All patients received either MMF or CYC, which was followed by AZA. IV CS (≤3g/d) were allowed by day 15 and oral CS (≤60 mg/d) were allowed with tapering over 10 weeks. Premed: IV MEP 100 mg, paracetamol 1 g, and AH | Diagnosis of SLE according to the ACR criteria, history of ANA positivity and active lupus nephritis (class III/IV) | Proportion of patients with a renal response at week 48 (complete renal response = normal serum creatinine and improvement in urinary protein: protein/crea­tinine ratio <0.5, partial renal response = serum creatinine ≤25% above baseline, and 50% improvement in urinary protein/crea­tinine and if urinary protein/crea­tinine ratio >3.0, then urinary protein/crea­tinine ratio <3.0, nonresponse = no complete or partial response) | Safety | Overall renal response: 66.7% renal response | See AEs | 109 patients (86.5%) | 34 patients (27.2%) with at least one SAE. 6 deaths: acute myeloid leukemia, acute myocar­dial infarc­tion, cardiac failure, cardio­respira­tory arrest, myocar­dial infarc­tion, pulmo­nary embolism |
|  |  | 47  (ITT 127) |  | 1000 mg on days 1 and 15, followed by a single infusion at week 16 and every 16 weeks thereafter | IV | Placebo |  |  |  | Safety | Overall renal response: 67.1% | See AEs | 102 patients (80.3%) |  |

| **Ofatumumab (OFA)** | | | | | | | | | | | | | | |
| --- | --- | --- | --- | --- | --- | --- | --- | --- | --- | --- | --- | --- | --- | --- |
| **ANCA-associated vasculitis (AAV)** | | | | | | | | | | | | | | |
| **Source** | **Patients treated** | **Com­pleting verum** | **Com­pleting control** | **OFA** | **Route** | **Control** | **Concomi­tant therapy** | **Inclusion criteria** | **Primary endpoint (1°)** | **Secondary endpoint (2°)** | **# Verum reaching 1°** | **# Verum reaching 2°** | **AEs** | **SAEs** |
| Mc Adoo et al., 2016 | 8 | 8 | No | 700 mg on days 0 and 14 | IV | No | Low-dose CYC IV (10 mg/kg) on days 0 and 14 and every 14 days thereafter, followed by maintenance therapy with AZA or MMF. Oral prednisolone (1 mg/kg) was started on day 0 with tapering to a dose of 10 mg by week 13. All patients received co-trimoxazole for 3 months, proton pump inhibitors and bone protection with calcium and vitamin D3 | Diagnosis of AAV | No primary endpoint defined | No secondary endpoint defined | Clinical remission was achieved in all patients by 3 months (defined as BVAS of 0 or BVAS ≤5 if persistent hematuria or proteinuria in the presence of stable or improving renal function). This was associated with a reduced acute phase protein response and the ability to taper CS. No clinical relapse during first year. One patient with eosinophilic granulomatosis with polyangiitis experienced 2 minor relapses at 15 and 21 months | No information | One patient with minor infusion reaction, 3 patients with infections (3 urinary tract infec­tions, 1 upper respirato­ry tract infection, 1 lower respirato­ry tract infection) and one patient with a transient episode of neutro­penia without infection |  |
| **Membranous nephropathy** | | | | | | | | | | | | | | |
| **Source** | **Patients treated** | **Com-pleting verum** | **Com-pleting control** | **OFA** | **Route** | **Control** | **Concomi-tant therapy** | **Inclusion criteria** | **Primary endpoint**  **(1°)** | **Secondary endpoint**  **(2°)** | **# Verum reaching 1°** | **# Verum reaching 2°** | **AEs** | **SAEs** |
| Podestà et al., 2019 | 3 | 3 | No control | Patient 1: 3 cycles of double-filtration plasmapheresis (DFPP) performed every other day followed by OFA. Patient 2: 100 mg OFA IV followed by 3 cycles of DFPP after 6 days.  Patient 3: 100 mg OFA IV followed by 4 cycles of DFPP after 5 days | IV | No control | Unknown | Diagnosis of anti-phospholipase A_2_-receptor-positive membranous nephropathy | No predefined primary endpoint | No predefined secondary endpoint | Patient 1: progression to end-stage renal disease with persistent nephrotic syndrome despite therapy.  Patient 2: transient decrease in anti-PLA_2_R antibodies but persistent nephrotic range proteinuria.  Patient 3: decrease in anti-PLA_2_R titer with partial remission of nephrotic syndrome | – | Unknown | Unknown |
| **Multiple sclerosis** | | | | | | | | | | | | | | |
| **Source** | **Patients treated** | **Com­pleting verum** | **Com­pleting control** | **OFA** | **Route** | **Control** | **Concomi­tant therapy** | **Inclusion criteria** | **Primary endpoint (1°)** | **Secondary endpoint (2°)** | **# Verum reaching 1°** | **# Verum reaching 2°** | **AEs** | **SAEs** |
| Sorensen et al., 2012 | 38 | 8  (ITT 8) | 4  (ITT 4) | OFA 100 mg twice two weeks apart with a switch to placebo after 24 weeks | IV | Placebo two weeks apart with a switch to OFA 100 mg after 24 weeks | Premed: paracetamol, AH and CS | Diagnosis of RRMS meeting McDonald 2005 criteria, aged 18-55 years, EDSS of 0 to 5, neurologi­cally stable, no evidence of relapse for ≥30 days, one of the following: ≥2 confirmed relapses within 24 months, ≥1 confirmed relapse within 12 months or 1 confirmed relapse 12 to 24 months before screening and ≥1 documen­ted T1 GdE lesion on MRI performed within 12 months. Exclusion criteria: PPMS, secondary progressive MS, treatment with lymphocyte-depleting therapies, mito­xantrone, CYC, or anti-CD20 agents at any time or glatiramer acetate or IFN-β-1a within the last 3 months or other immunosuppressive or immuno­modulatory agents within 6 months, plasmaphe­resis within the last 2 months, CS, adrenocor­ticotropic hormone or live vaccine within the last month | Safety as assessed through number of AEs, MRI, and clinical laboratory tests | Cumulative number of new GdE lesions, T2 lesions, and T1 hypointense lesions. Proportion of relapse-free patients, relapse rate, and change in EDSS from baseline to week 24 and from week 24 to 48 | Weeks 0-24: 8 patients developed at least one AE; weeks 24-48: 3 patients with at least one AE | Weeks 0-24: relative reduction in number of new T1 GdE lesions (p<0.001), total number of T1 GdE lesions (p<0.001), and new and/or enlarging T2 lesions (p<0.001). Overall: no clinically signifi­cant changes in EDSS or MSFC scores. 5 patients (19%) relapsed during the first 24 weeks | See primary endpoint | No SAEs |
|  |  | 10  (ITT 11) | 3  (ITT 4) | OFA 300 mg twice two weeks apart with a switch to placebo after 24 weeks |  | Placebo two weeks apart with a switch to OFA 300 mg after 24 weeks |  |  |  |  | Weeks 0-24: 10 patients developed at least one AE; weeks 24-48: 4 patients with at least one AE |  |  | 1 patient with an SAE during weeks 0-24 |
|  |  | 7  (ITT 7) | 4  (ITT 4) | OFA 700 mg twice two weeks apart with a switch to placebo after 24 weeks |  | Placebo two weeks apart with a switch to OFA 700 mg after 24 weeks |  |  |  |  | Weeks 0-24: 7 patients developed at least one AE; weeks 24-48: 4 patients with at least one AE |  |  | No SAEs |
| Bar-Or et al., 2018 (MIRROR-Study) | 231 | 30  (ITT 34) | 63  (ITT 67) | OFA 3 mg every 12 weeks | SC | Placebo | Premed: paracetamol, AH | Diagnosis of RRMS according to the revised McDonald criteria, at least one confirmed relapse within the previous year or at least 2 confirmed relapses within the previous 2 years or at least one relapse in the previous 2 years with a GdE brain lesion within the past year, 18-55 years old, EDSS of 0-5.5. Exclusion criteria: pregnancy, prior use of experimental agents, monoclonal antibodies (except natalizu­mab) or immunosuppressive agents | Cumulative number of new GdE brain lesions at week 12 | Proportion of relapse-free patients, EDSS, MFIS, cumulative number of new GdE lesions at week 24, cumulative number and total volume of new and new plus persisting GdE lesions and/or newly enlarging T2 lesions, and T1-hypointense lesions at week 12 and 24 | 65% reduction in the mean rate of cumulative new GdE lesions (p<0.001) | No signifi­cant differen­ces concer­ning the EDSS and relapse rates | 24 patients (71%) with an AE | 0 patients with an SAE. No deaths |
|  |  | 30  (ITT 32) |  | OFA 30 mg every 12 weeks |  |  |  |  |  |  |  |  | 23 patients (72%) with an AE | 0 patients with an SAE. No deaths |
|  |  | 32  (ITT 34) |  | OFA 60 mg every 12 weeks |  |  |  |  |  |  |  |  | 22 patients (65%) with an AE | 1 patient (3%) with an SAE. No deaths |
|  |  | 57  (ITT 64) |  | OFA 60 mg every 4 weeks |  |  |  |  |  |  |  |  | 52 patients (81%) with an AE | 4 patients (6%) with an SAE. No deaths |
| Hauser et al., 2020 (ASCLEPIOS I) | 927 | 416 (ITT 465) | 376 (ITT 462) | 20 mg OFA SC loading dose on days 1, 7, and 14, followed by injections every 4 weeks | SC / oral | 14 mg teriflunomide orally every day | Premedication with CS, acetaminophen and AH were allowed | Diagnosis of RRMS or secondary progressive MS according to 2010 revised McDonald criteria, EDSS of 0-5.5, age 18-55 years, 1 relapse in the year before screening, 2 relapses in the 2 years before screening, or at least 1 lesion on MRI with a stable condition for at least 1 month before randomization. Exclusion criteria: PPMS, neuromyelitis optica disorder, pregnancy, lactation, immunodeficiency, progressive multifocal leukoencephalopathy, HIV infection, hepatitis, syphilis, tuberculosis, pretreatment with specific drugs | Annualized relapse rate | Disability worsening at 3 and 6 months, disability improvement at 6 months, number of GdE lesion on T1, number of T2 lesions and annual rate of brain-volume loss | 0.11 versus 0.22 (p<0.001) | Meta-analysis of pooled trials:  Disability worsening at 3 months: 10.9% versus 15% (p=0.002).  Disability worsening at 6 months: 8.1% versus 12% (p=0.01).  Disability improvement at 6 months: 11% versus 8.1% (p=0.09). Significant reduction in T1-weighted GdE and T2 lesions in both trials (p<0.001). No significant difference in brain volume loss | 791/946 (83.6%) AEs in OFA versus 788/936 (84.2%) AEs in teriflunomide | 9.1% of patients receiving OFA experienced an SAE versus 7.9% of patients receiving teriflunomide |
| Hauser et al., 2020 (ASCLEPIOS II) | 955 | 397 (ITT 481) | 389 (ITT 474) | 20mg OFA SC loading dose at days 1, 7, and 14 followed by injections every 4 weeks. | SC / oral | 14mg teriflunomide orally every day |  |  | Annualized relapse rate | Disability worsening at 3 and 6 months, disability improvement at 6 months, number of GdE lesion on T1, number of T2 lesions and annual rate of brain volume loss | 0.10 versus 0.25 (p<0.001) |  |  |  |

| **Rheumatoid arthritis (RA)** | | | | | | | | | | | | | | |
| --- | --- | --- | --- | --- | --- | --- | --- | --- | --- | --- | --- | --- | --- | --- |
| **Source** | **Patients treated** | **Com­pleting verum** | **Com­pleting control** | **OFA** | **Route** | **Control** | **Concomi­tant therapy** | **Inclusion criteria** | **Primary endpoint (1°)** | **Secondary endpoint (2°)** | **# Verum reaching 1°** | **# Verum reaching 2°** | **AEs** | **SAEs** |
| Oster­gaard et al., 2010 | Part A: 39,  Part B: 225 | Part A: 12,  Part B: 58 | Part A:7, Part B: 55 | 300 mg twice two weeks apart | IV | Placebo | Stable MTX was allowed if taken for at least 12 weeks with a stable dose for at least 4 weeks. All other DMARDs had to be withdrawn. Stable oral CS (≤10 mg/d), NSAIDs, analgesics and one IA injection of CS in a single joint were allowed. Premed: AH IV and oral paracetamol. Later, oral CS were added the day before the infusion as well as IV prednisolone 100 mg 60-120 min before the infusion | Diagnosis of active RA according to the ACR criteria, disease duration ≥6 months, inadequate response to ≥1 DMARD | Part A: safety of 3 doses of OFA.  Part B: proportion of patients with an ACR improvement | DAS28, EULAR response criteria, duration and degree of B cell depletion, ADAs, and PK | Part A: 12 patients with at least one AE. In total: 59 patients with at least one AE | DAS 28: Part A -2.15, Part B -1.81. Moderate or good EULAR response: Part A 78%, Part B 70% (p<0.002) | See primary endpoint | 3 patients (5%) with at least one SAE in Part B. No SAEs in Part A |
|  |  | Part A: 10,  Part B: 57 |  | 700 mg twice two weeks apart | IV |  |  |  |  |  | Part A: 10 patients with at least one AE. In total: 58 patients with at least one AE | DAS 28: Part A  -2.15, Part B  -1.81. Moderate or good EULAR response: Part A 78%, Part B 70% (p<0.002) | See primary endpoint |  |
|  |  | Part A: 10,  Part B: 54 |  | 1000 mg twice two weeks apart | IV |  |  |  |  |  | Part A: 9 patients with at least one AE. In total: 54 patients with at least one AE | DAS 28: Part A -2.15, Part B -1.81. Moderate or good EULAR response: Part A 78%, Part B 70% (p<0.002) | See primary endpoint |  |
| Taylor et al., 2011 | 260 | 117  (ITT 129) | 121  (ITT 131) | 700 mg twice two weeks apart | IV | Placebo | All patients received stable MTX. Analgesics, NSAIDs, oral CS (≤10 mg/d) and one IA CS injection in one joint per 6-month period were allowed. Premed: AH, oral paracetamol, and MEP 100 mg IV | Diagnosis of active RA according to the ACR criteria, disease duration ≥6 months duration, inadequate response to MTX despite treatment for at least 12 weeks with stable doses for at least 4 weeks | Proportion of patients achieving an ACR20 response at week 24 | Proportion of patients achieving an ACR50/70 response, a good or moderate EULAR response, mean change in DAS28, 36-item short form health survey version 2.0, FACIT-F, HAQ-DI, AEs, and immunogenicity were assessed | 64/129 patients reached an ACR20 response | 35/129 reached an ACR50 response and 17/129 reached and ACR70 response | 116 patients (89%) with at least one AE | 4 patients (3%) had at least one SAE (4 SAEs in 4 patients: bacterial gastro­enteritis, pneumo­nia, myocar­dial infarc­tion, ischemic stroke). No deaths |
| Kurrasch et al., 2013 | 35 | 3  (ITT 4) | 6  (ITT 8) | 0.3 mg once | SC | Placebo | All patients received stable MTX. Stable dose of oral prednisolone (≤10 mg/d) was allowed. Premed: oral paracetamol and oral AH | Diagnosis of RA according to the ACR criteria, disease duration ≥6 months, previous treatment with MTX for at least 12 weeks with a stable dose over the last 4 weeks, BMI <35, corrected QT interval <450 ms, negative screening chest radiograph for lung infection | Safety and tolerability of SC OFA by assessing incidence and severity of AE, clinical laboratory measures, and vital signs | Minimum dose of OFA resulting in depletion of peripheral blood B cells, time to repletion, PK, incidence of ADAs, and biomarkers of disease activity and immune status | 4 patients with at least one AE | No ADAs. Plasma concentrations after one single administration near or below LLQ. No notable pattern for change in median systemic markers IL-6, SAA, RF, ESR or CRP over time | See primary endpoint | No SAE in placebo treated patients |
|  |  | 6  (ITT 6) |  | 3 mg once | SC |  |  |  |  |  | 5 patients with at least one AE | No ADAs. Plasma concen­trations after one single admini­stration near or below LLQ. B cell depletion achieved by 1/6 patients. No notable pattern for change in IL-6, SAA, RF, ESR or CRP over time | See primary endpoint |  |
|  |  | 4  (ITT 8) |  | 30 mg once | SC |  |  |  |  |  | 8 patients with at least one AE | No ADAs. Mean half-life time from 5.20 to 6.83 days. B cell depletion achieved by 7/8 patients. No notable pattern for change in IL-6, SAA, RF, ESR or CRP over time | See primary endpoint |  |
|  |  | 6  (ITT 6) |  | 60 mg once | SC |  |  |  | Safety and tolerability of SC OFA by assessing incidence and severity of AE, clinical laboratory measures, and vital signs | Minimum dose of OFA resulting in depletion of peripheral blood B cells, time to repletion, PK, incidence of ADAs, and biomarkers of disease activity and immune status | 4 patients with at least one AE | No ADAs. Mean half-life time from 5.20 to 6.83 days. B cell depletion achieved by all patients. No notable change in IL-6, SAA, RF, ESR or CRP over time | See primary endpoint |  |
|  |  | 3  (ITT 3) |  | 100 mg once | SC |  |  |  | Safety and tolerability of SC OFA by assessing incidence and severity of AE, clinical laboratory measures, and vital signs | Minimum dose of OFA resulting in depletion of peripheral blood B cells, time to repletion, PK, incidence of ADAs, and biomarkers of disease activity and immune status | 3 patients with at least one AE | No ADAs. Mean half-life time from 5.20 to 6.83 days. B cell depletion achieved by all patients. No notable change in IL-6, SAA, RF, ESR or CRP over time | See primary endpoint |  |
| Quattrocchi et al., 2016 (OFA110634): not published | 169 | ITT 86 | ITT 83 | 700 mg twice two weeks apart | IV | Placebo | Stable MTX was allowed. All other DMARDs had to be withdrawn. Analgesics, NSAIDs and one IA CS injection in one joint per 6-month period were allowed. Premed: oral AH, oral paracetamol and IV MEP | Diagnosis of active RA according to the ACR criteria, disease duration ≥6 months duration, inadequate response to TNF inhibitors (DAS ≥3.2), MTX therapy for at least 12 weeks with stable doses for at least 4 weeks | Proportion of patients achieving an ACR20 response at week 24 | ACR50/70 response, DAS28, EULAR response | No information | No informa­tion | 72 patients (84%) with at least one AE | 6 patients (7%) with at least one SAE. No deaths |
| Quattrocchi et al., 2016 (Exten­sion trial of Oster­gaard et al., 2010) (OFA111752): not published | 92 | ITT 68 | ITT 24 | 300 mg twice two weeks apart | IV | No control | Stable MTX was allowed if taken for at least 12 weeks with a stable dose for at least 4 weeks. All other DMARDs had to be withdrawn. Stable oral CS (≤10 mg/d), NSAIDs, analgesics and one IA injection of CS in a single joint were allowed. Premed: AH IV and oral paracetamol. Later, oral CS were added the day before the infusion as well as IV prednisolone 100 mg 60-120 min before the infusion | Diagnosis of active RA according to the ACR criteria, disease duration ≥6 months, inadequate response to ≥1 DMARD | Time to treatment withdrawal defined as the time from first infusion of OFA until date of treatment withdrawal | Minimum DAS28 score over 16-24 weeks, EULAR response at 16, 20, or 24 weeks, time to first retreatment | No information | No informa­tion | 70/92 patients (76%) had at least on AE |  |

| **Systemic lupus erythematosus (SLE)** | | | | | | | | | | | | | | |
| --- | --- | --- | --- | --- | --- | --- | --- | --- | --- | --- | --- | --- | --- | --- |
| **Source** | **Patients treated** | **Com­pleting verum** | **Com­pleting control** | **OFA** | **Route** | **Control** | **Concomi­tant therapy** | **Inclusion criteria** | **Primary endpoint (1°)** | **Secondary endpoint (2°)** | **# Verum reaching 1°** | **# Verum reaching 2°** | **AEs** | **SAEs** |
| Haarhaus et al., 2016 | 4 | 1 | No control | 300 mg on day 0 and 700 mg on day 15 with a retreatment after 14 months with 700 mg twice 3 weeks apart | IV | No control | Prednisolone 10 mg/d, MMF 2 g/d and antimalarials 200 mg/d | Diagnosis of refractory lupus nephritis with initial response to rituximab, but develop­ment of infusion reactions | No primary endpoint defined | No secondary endpoint defined | After 2 infusions of OFA disease remained active and OFA was again given after 14 months. U-ACR (Urine albumine-to-creatinine ratio) is 19 mg/ mmol 18 months after the last infusion | No informa­tion | No AE observed |  |
|  |  | 1 | No control | 100 mg on day 0, 600 mg on day 1 and 700 mg after 2 weeks. Re-treatment after 3 months | IV | No control | Prednisolone 30 mg/d |  | No primary endpoint defined | No secondary endpoint defined | Patient initially responded to treatment but relapsed after 3 months. OFA re-administered and U-ACR decreased to 108 mg/mmol 5 months later. However, patient experienced a severe non-renal flare and OFA 700 mg was given, which resulted in widespread urticaria. OFA was discontinued | No informa­tion | Wide­spread urticaria |  |
|  |  | 1 | No control | 700 mg on days 0 and 14 and every 6 months thereafter | IV | No control | Prednisolone 5 mg/d, cyclosporin A 200 mg/d |  | No primary endpoint defined | No secondary endpoint defined | 3 years after initiation of OFA, U-ACR is 32 mg/mmol | No informa­tion | No AEs observed |  |
|  |  | 1 | No control | 200 mg at day 0, 700 mg on days 1 and at 2 weeks | IV | No control | Prednisolone 10 mg/d, antimalarials 400 mg/d |  | No primary endpoint defined | No secondary endpoint defined | Combined with cyclosporine A treatment OFA resulted in a U-ACR of 3 mg/mmol | No informa­tion | No AEs observed |  |

| **Ublituximab** | | | | | | | | | | | | | | |
| --- | --- | --- | --- | --- | --- | --- | --- | --- | --- | --- | --- | --- | --- | --- |
| **Multiple sclerosis** | | | | | | | | | | | | | | |
| **Source** | **Patients treated** | **Com­pleting verum** | **Com­pleting control** | **Ublituximab** | **Route** | **Control** | **Concomi­tant therapy** | **Inclusion criteria** | **Primary endpoint (1°)** | **Secondary endpoint (2°)** | **# Verum reaching 1°** | **# Verum reaching 2°** | **AEs** | **SAEs** |
| Fox et al., 2021 | 60 | 49 (ITT 48)  Including cross-over from placebo-group. | 13 | Ublituximab on days 1, 15 and week 24.  Day 1: 150mg over 1, 2, 3 or 4 h.  Day 15: 450mg over 1, 1.5 or 3 h or 600mg over 1h.  Week 24: 450mg over 1 or 1.5h or 600mg over 1h. | IV | Placebo | Premed: oral AH and oral CS | Relapsing MS as defined per 2010 McDonald Criteria, EDSS 0-5.5, age 18-55 years, neurologically stable for 30 days or more, at least 2 relapses during the previous 2 years or 1 relapse within 1 year. Exclusion criteria: prior treatment with anti-CD20 or other B-cell-directed therapy or with alemtuzumab within the last year, use of fingolimod or natalizumab within 90 days, use of glatiramer acetate, IFN-beta, dimethyl fumarate, or glucocorticoids within 30 days, disease duration > 10 years. | Responder rate (proportion of patients with at least 95% depletion of CD19+ B-cells) | Change in GdE T1 and T2 lesions, annualized relapse rate, proportion of relapse free patients | 100% (p<0.001) | GdE T1 lesions: no new or persisting lesions during the 48 weeks study duration.  GdE T2 lesions: volume depletion of 10.6% from baseline to week 48. 7 patients developed new T2 lesions.  Annualized relapse rate: 93% of all patients remained relapse free during the 48 weeks. | Unknown | 1 SAE (fatigue) |
| **Neuromyelitis optica spectrum disorder** | | | | | | | | | | | | | | |
| **Source** | **Patients treated** | **Com­pleting verum** | **Com­pleting control** | **Ublituximab** | **Route** | **Control** | **Concomi­tant therapy** | **Inclusion criteria** | **Primary endpoint (1°)** | **Secondary endpoint (2°)** | **# Verum reaching 1°** | **# Verum reaching 2°** | **AEs** | **SAEs** |
| Mealy et al., 2019 | 5 | 5 | No control | 450mg once on days 1-5. | IV | No control | 1000mg IV-MP on days 1-5. Plasmapheresis from day 5 on if improvement with CS alone was not sufficient. 3 of 5 patients received baseline treatment with MMF, or RTX | Diagnosis of NMOSD according to the 2015 International Panel for NMO Diagnosis criteria, age 18-65 years, new neurologic symptoms with contrast-enhancing lesion on MRI. | Safety | Disability, Remission, and B cell counts | There was 1 SAE (leukopenia). 3/5 patients with a headache and body pain | 4 of 5 patients experienced a b cell depletion. 2 patients developed relapses within 90 days. | See primary endpoint | See primary endpoint |

| **Veltuzumab (VEL)** | | | | | | | | | | | | | | |
| --- | --- | --- | --- | --- | --- | --- | --- | --- | --- | --- | --- | --- | --- | --- |
| **Immune thrombocytopenia (ITP)** | | | | | | | | | | | | | | |
| **Source** | **Patients treated** | **Com­pleting verum** | **Com­pleting control** | **VEL** | **Route** | **Control** | **Concomi­tant therapy** | **Inclusion criteria** | **Primary endpoint (1°)** | **Secondary endpoint (2°)** | **# Verum reaching 1°** | **# Verum reaching 2°** | **AEs** | **SAEs** |
| Liebman et al., 2013 | 41 | 3 | No control | 80 mg twice 2 weeks apart | IV | No control | Stable doses of prednisone (≤20 mg/d) and danazol were allowed. Premed: oral antipyretics and AH | Diagnosis of primary ITP as defined by the American Society of Hematolo­gy guidelines, with or without splenec­tomy, failure to ≥1 standard ITP therapy, platelet count <30x10^9^/L on 2 occasions at least 1 week apart | No information. Objective of study: evaluation of safety, efficacy, PD, PK, and immuno­genicity. Objective response defined as platelet count ≥30x10^9^/L measured twice at least one week apart with at least two-fold increase from baseline. Objective responses categorized as complete if platelet count ≥100x10^9^/L and as partial if platelet count lower | No information | 4 patients with objective response, 2 patients with complete response | Median time to relapse: 8 months, with 10 patients relapse free for >1 year. B cell depletion and recovery compa­rable across VEL doses. IgG, IgA, and T cells no consistent change from baseline. IgM decreased consis­tently, but no clinical signifi­cance. 9 patients showed elevated ADAs (1 already positive at baseline) | 5 AEs: all transient Grade 1-2 infusion reactions (2 throat discom­fort, 1 fever, 1 body aches, 1 nausea) |  |
|  |  | 3 | No control | 120 mg twice 2 weeks apart | IV | No control |  |  |  |  |  |  |  |  |
|  |  | 1 | No control | 200 mg twice 2 weeks apart | IV | No control |  |  |  |  |  |  |  |  |
|  |  | 9 | No control | 80 mg twice 2 weeks apart | SC | No control |  |  |  |  | 17 patients with objective response, 9 patients with complete response |  | 24 AEs: 17 local injection site reactions (pain, burning or discomfort, erythema, swelling, bruising, cellulitis) and 11 constitu­tional symp­toms (myalgia, fever, chills, headache, nausea, pruritus, vomiting, fatigue, cold-like symp­toms). One Grade 3 AE: genera­lized pain not otherwise specified |  |
|  |  | 10 | No control | 160 mg twice 2 weeks apart | SC | No control |  |  |  |  |  |  |  |  |
|  |  | 15 | No control | 320 mg twice 2 weeks apart | SC | No control |  |  |  |  |  |  |  |  |
| Liebman et al., 2016 | 50 | 9 | No control | 80 mg twice 2 weeks apart | SC | No control | Prednisone ≤20 mg/d and danazol were allowed if given in a stable dose. Premed with paracetamol, diphen­hydramine or benzodia­zepines was possible at the investigators' discretion | Diagnosis of primary ITP as defined by the American Society of Hematolo­gy guidelines, with or without splenec­tomy, failure to ≥1 standard ITP therapy, platelets <30x10^9^/L on 2 occasions at least 1 week apart | No information. Objective response defined as platelet count ≥30x10^9^/L measured twice at least one week apart with at least two-fold increase from baseline. Objective responses categorized as complete response if platelet count ≥100x10^9^/L and as partial if platelet count lower. Analysis of safety, PD, PK, and immunoge­nicity | No information | 23/47 patients with objective response, including 15/47 with complete response | Median time to response: 26 days. Median time to relapse: 1.3 years. Response duration increased with higher VEL doses. Patients with complete response had longer response duration. Bleeding reduction in all treatment groups without evidence of a dose response. B cell depletion compa­rable across doses. B cell recovery slower in higher doses. T cells and Igs no consistent change from baseline. 11 patients had elevated ADAs (2 patients already positive at baseline) | 7 patients (78%) with at least one AE |  |
|  |  | 10 | No control | 160 mg twice 2 weeks apart | SC | No control |  |  |  | No information |  |  | 8 patients (80%) with at least one AE |  |
|  |  | 15 | No control | 320 mg twice 2 weeks apart | SC | No control |  |  |  | No information |  |  | 9 patients (60%) with at least one AE |  |
|  |  | 16 | No control | 320 mg weekly for 4 weeks | SC | No control |  |  |  | No information |  |  | 15 patients (94%) with at least one AE |  |

**Abbreviations and explanations**: AAV, ANCA-associated vasculitis; ACR, American College of Rheumatology; ADAs, anti-drug antibodies; AE, adverse event; AH, antihistamine; ANA, antinuclear antibody; ANCA, anti-neutrophil cytoplasmic antibody; AZA, azathioprine; BVAS, Birmingham Vasculitis Activity Score; BMI, body mass index; CRR, complete renal response; CRP, C-reactive protein; CS, corticosteroid(s); CYC, cyclophosphamide; DAS28, 28-joint disease activity score; DAS28-ESR, DAS28–erythrocyte sedimentation rate; DFPP, double-filtration plasmapheresis; DMARD, disease-modifying antirheumatic drug; EDSS, expanded disability status scale; ESR, erythrocyte sedimentation rate; EULAR, European League Against Rheumatism; FACIT-F, Functional Assessment of Chronic Illness Therapy-Fatigue; GdE, gadolinium-enhancing; HAQ-DI, health assessment questionnaire–disability index; IA, intraarticular(ly); IFN, interferon; Ig, immunoglobulin; IM, intramuscular; IRR, infusion-related reaction; ITP, immune thrombocytopenia; ITT, intention-to-treat; IV, intravenous(ly); LEF, leflunomide; LLQ, lower limit of quantification; MEP, methylprednisolone; MFIS, Modified Fatigue Impact Scale; MMF, mycophenolate mofetil; MS, multiple sclerosis; MSFC, Multiple Sclerosis Functional Composite; mTSS, Modified total Sharp/van der Heijde score; MTX, methotrexate; NSAID, non-steroidal antirheumatic drug; OBI, obinutuzumab; OFA, ofatumumab; OCR, ocrelizumab; ORR, overall renal response; paracetamol = acetaminophen; PD, pharmacodynamics; PK, pharmacokinetics; PLA_2_R, phospholipase A_2_ receptor; PPMS, primary progressive multiple sclerosis; Premed, premedication; RA, rheumatoid arthritis; RF, rheumatoid factor; RRMS, relapsing-remitting multiple sclerosis; SAA, serum amyloid A; SAE, serious adverse event; SC, subcutaneous(ly); SLE, systemic lupus erythematosus; SHS, Sharp/van der Heijde score; TNF, tumor-necrosis factor; U-ACR, urine albumine-to-creatinine ratio; VEL, veltuzumab.
